# Sublethal concentrations of antibiotics enhance transmission of antibiotic resistance genes in environmental *Escherichia coli*

**DOI:** 10.3389/fmicb.2025.1675089

**Published:** 2025-10-23

**Authors:** Charlotte J. Gray-Hammerton, Steven P. Hooton, Kirsty Sands, Timothy R. Walsh, Claudia Orbegozo Rubio, Edward A. R. Portal, Christopher Hudson, Dov J. Stekel, Christine E. R. Dodd, Jon L. Hobman

**Affiliations:** ^1^School of Biosciences, University of Nottingham, Sutton Bonington Campus, Sutton Bonington, United Kingdom; ^2^Department of Biology, Ineos Oxford Institute for Antimicrobial Research (IOI), University of Oxford, Oxford, United Kingdom; ^3^Department of Medical Microbiology, Division of Infection and Immunity, Cardiff University, Cardiff, United Kingdom; ^4^School of Veterinary Science and Medicine, University of Nottingham, Sutton Bonington Campus, Sutton Bonington, United Kingdom; ^5^Department of Mathematics and Applied Mathematics, University of Johannesburg, Johannesburg, South Africa

**Keywords:** AMR, ESBL, dairy, ISEcp1, transposition, ST2325

## Abstract

Third-generation cephalosporin-resistant Enterobacterales are ranked second on the World Health Organisation (WHO)’s Bacterial Priority Pathogens List. Amongst them, extended-spectrum β-lactamase-producing *Escherichia coli* (ESBL-Ec) are used by the WHO as sentinel organisms to monitor the spread of antibiotic resistance worldwide and are often associated with mobilisable multidrug resistance (MDR). However, we know less about how ESBL-producing genes spread in environmental *E. coli*. This study investigates how the *bla*_CTX-M-15_ gene from ESBL-Ec isolated on a UK dairy farm could transfer between strains. For this study, 39 *E. coli* were isolated from a single dairy farm over 4 months, using cefotaxime-supplemented selective media. All had similar antibiotic susceptibility test phenotypes, and PCR, whole genome sequencing (WGS), and resistance gene transmission experiments demonstrated they were all closely related. *In silico* multi-locus sequence typing and single-nucleotide polymorphism analysis showed that all 39 strains were Sequence Type 2325, but plasmid carriage differed. In total, 35 of the 39 ESBL-Ec strains were multidrug resistant, displaying *bla*_CTX-M_ type cephalosporin resistance and resistance to fluoroquinolones and tetracyclines. WGS confirmed all 39 isolates carried the IS*Ecp1* mobile genetic element carrying the *bla*_CTX-M-15_ ESBL-producing gene, and the *qnrS1* partial quinolone resistance gene in the chromosome. A total of 35 strains also carried *tetAR* within this IS*Ecp1* element. We found that sub-lethal levels of ampicillin, cloxacillin, and ceftazidime could enhance the transfer of IS*Ecp1 bla*_CTX-M-15_ from the chromosome of these dairy farm strains into endogenous self-transmissible plasmids, which can themselves then transfer into and confer phenotypic antibiotic resistance in a recipient *E. coli* K-12 strain. In conclusion, we observed not only clonal dissemination of these environmentally occurring ESBL-producing strains within the farm environment but also showed experimentally that these strains had the ability to mobilise their ESBL producing genes, and that these and other resistance genes can be acquired or lost on transfer. This shows the importance of longitudinal monitoring of antibiotic resistance, especially in places with high prevalence or selective pressure for resistant bacteria.

## 1 Introduction

The World Health Organisation (WHO) has identified third-generation cephalosporin-resistant Enterobacterales as critical priority pathogens (WHO, 2024) and uses extended spectrum β-lactamase producing *Escherichia coli* (ESBL-Ec) as sentinel organisms in its Tricycle protocol for global AMR surveillance (WHO, 2021). ESBL-Ec is an important antimicrobial-resistant (AMR) organism due to its resistance to penicillin and cephalosporin antibiotics and because of the rapid worldwide spread of *bla*_CTX-M_ type ESBLs (Cantón and Coque, 2006; Cantón et al., 2012), including in multi-drug-resistant clinical Enterobacterial strains (Ibrahim et al., 2016). The association of ESBL-producing *bla*_CTX-M_ genes with mobile genetic elements such as plasmids and transposons has led to transmission and acquisition of resistance to human critical cephalosporin antibiotics in Enterobacterales in a wide range of human and animal environments, including in high-intensity livestock production such as dairy farms (Eckert et al., 2004; Liebana et al., 2013; Irrgang et al., 2017).

The insertion sequence (IS) element, IS*Ecp1,* has played a key role in the acquisition and dissemination of *bla*_CTX-M_ from the posited original source of this resistance, the soil-associated bacterium *Kluyvera ascorbata* (Humeniuk et al., 2002; Rossolini et al., 2008; Zong et al., 2010; Bevan et al., 2017; Afema et al., 2018). IS*Ecp1* assists DNA adjacent to where it has inserted in chromosomes or plasmids to move to other places in the genome, via a one-ended transposition mechanism that produces 5-bp target site duplications at the point the element inserts (Poirel et al., 2005; Kieffer et al., 2020). IS*Ecp1* is defined by and flanked by 14-bp Inverted Repeat left (IR_L_) and degenerate right IR sequences (IR_R_). Due to the recognition of an imperfect IR_R_ sequence by IS*Ecp1,* it can mobilise different-sized transposition units (Poirel et al., 2005) because downstream genes can be collected or lost as mobilisation takes place (Zong et al., 2010). IS*Ecp1* also provides the −35 and −10 promoter sequences for high-level expression of *bla*_CTX-M_ (Poirel et al., 2005; Zong et al., 2010).

Previous studies have shown successful transposition of a cloned IS*Ecp1* in *K. ascorbata* from a chromosomal location into a plasmid that had been introduced into the strain, with IS*Ecp1* transposition enhanced in the presence of several β-lactam antibiotics (Lartigue et al., 2006; Nordmann et al., 2008). A further study by Hamamoto et al. (2020) demonstrated the transposition of IS*Ecp1* carrying *bla*_CTX-M-14_, from a plasmid construct location to a chromosomal location within an experimental *E. coli* strain. To the best of our knowledge, no published studies have explored transposition of a naturally occurring chromosomally encoded IS*Ecp1* or have addressed the question of whether endogenous plasmids in the host strain could then transfer mobilised IS*Ecp1* into a recipient strain and thus provide phenotypic cephalosporin resistance to it. Although previous studies have demonstrated enhanced transposition of IS*Ecp1* in the presence of ceftazidime, cefotaxime, and piperacillin (Lartigue et al., 2006; Nordmann et al., 2008), the presence of sub-lethal levels of other β-lactam antibiotics, such as those that might be encountered in therapeutically-treated dairy cattle, needs to be examined for their potential in promoting resistance dissemination via this mechanism.

The aim of this study was to investigate the carriage, dissemination, and transmission of *bla*_CTX-M_ resistance in *E. coli* isolates from a UK dairy farm. Previously described Antimicrobial Susceptibility Testing (AST) data (Baker et al., 2022) of a collection of 811 confirmed *E. coli* dairy farm strains were used to select a subset of 39 ESBL isolates with a *bla*_CTX_ type resistance phenotype. We used PCR to confirm the presence of IS*Ecp1* and *bla*_CTX-M-15_ in the 39 ESBL-Ec, which were then fully characterised by whole genome sequencing (WGS). We then interrogated whether the IS*Ecp1* element in these strains could transpose *bla*_CTX-M-15_ from the chromosome into their endogenous conjugative plasmids and then transfer cefotaxime resistance to a recipient *E. coli* strain. Next, we investigated whether the presence of sub-lethal levels of β-lactam antibiotics, such as those that could be encountered in therapeutically treated dairy cattle, might promote transfer of IS*Ecp1 bla*_CTX-M-15_ resistance. In summary, we characterised a group of naturally occurring ESBL-Ec isolated over a 4-month period from a single dairy farm and then demonstrated that they can transfer antibiotic resistance into other *E. coli* strains.

## 2 Materials and methods

### 2.1 Bacterial strains and growth conditions

The 39 *E. coli* dairy farm isolates were chosen from a larger collection of 811 *E. coli* strains, taken as part of an integrated AMR study. Isolation and initial characterisation of strains are described in Baker et al. (2022) and Todman et al. (2024). Supplementary Table S1 shows the farm strains studied, date of isolation, sampling location, the selective media used for initial isolation, and the resistance profiles from the disc diffusion assay data supplied in Baker et al. (2022).

The 39 strains were selected on the basis of their ESBL type phenotype using the CLSI standard (CLSI, 2018) disc diffusion assays, which included resistance to the semi-synthetic penicillin, ampicillin (AMP), and to the cephalosporin, cefotaxime (CTX), but with susceptibility to the β-lactam/β-lactamase inhibitor combination amoxicillin/clavulanic acid (AMC) and to the cephamycin cefoxitin (FOX). A separate, but closely related ESBL isolate, EcoSL3110-774 (strain 774), with a similar antimicrobial susceptibility test (AST) profile, which was sequenced using PacBio as part of the dairy farm study (Baker et al., 2022), and it was also analysed in detail as part of this study. Strain 774 was found to contain an IS*Ecp1 bla*_CTX-M-15_ element, and as this was complete and within one contig, this allowed for a detailed genetic environment to be constructed; however, no plasmids were found in strain 774.

The kanamycin (KAN) and rifampicin (RIF)-resistant *E. coli* K-12 strain CV601 encoding green fluorescent protein (GFP) (Smalla et al., 2000) was used as the recipient in all conjugation assays.

For all PCR and genetic work and reviving strains for AST assays, all strains were revived on or in either solid or liquid Lysogeny Broth (LB) Miller media (Sigma-Aldrich, USA) and at 37°C overnight for between 18 and 20 h. When revived in LB, cultures were grown overnight for 18–20 h at 37 °C with agitation at 180 RPM (Medline Scientific™ ISF-7100 Floor Standing Incubator Shaker, Fisher Scientific, UK).

### 2.2 Minimum inhibitory concentration (MIC) agar dilution assays

The 39 isolates were further characterised phenotypically using agar dilution MIC assays according to CLSI guideline methods (CLSI, 2018) but using EUCAST susceptibility breakpoints (EUCAST, 2022) (where available). A panel of 25 antibiotics was used in the MIC assays. These are listed in Supplementary Table S2 along with the concentration ranges tested for each antibiotic, the breakpoint utilised, and full details relating to the antibiotic discs used by Baker et al. (2022) in disc diffusion tests listed in Supplementary Table S3.

### 2.3 DNA extraction and purification

Total DNA for PCR was isolated from *E. coli* strains using the simple boiling method as previously described (Wei, 2013). PCR products requiring Sanger sequencing were purified using a NEB T1030 Monarch^®^ PCR & DNA Cleanup kit according to the manufacturer’s instructions (NEB, USA). Genomic DNA (gDNA) extraction was conducted at the University of Cardiff, using a Qiagen QIAamp DNA Mini QIAcube kit using the QIAcube platform (QIAGEN, Germany) with an additional RNAase step. The gDNA was quantified using a Qubit v4.0 (Thermo Fisher Scientific, Loughborough, UK). The gDNA extracted was used to generate libraries for both the MiSeq (Illumina, USA) short read and MinION long read (Oxford Nanopore Technologies, UK) sequencing.

### 2.4 PCR

PCR experiments were used to determine the presence of IS*Ecp1* and *bla*_CTX-M_ genes in isolates with a CTX-M type antibiotic resistance profile, and to confirm the presence of *gfp* in CV601 transconjugants. DreamTaq Green Mastermix (ThermoFisher Scientific, Loughborough, UK) was used according to the manufacturer’s instructions. Oligonucleotides were synthesised by Eurofins Genomics (Ebersberg, Germany) and are listed in Table 1. Oligonucleotides were used at a final working concentration of 10 pmol μl^−1^. All PCR conditions consisted of an initial denaturation at 95 °C for 5 min, followed by 30 cycles of 94 °C for 1 min, an annealing temperature (T_A_), annealing time, and extension at 72 °C specific to each gene (Table 1), and a final incubation at 72 °C for 10 min. PCR amplification of the *bla*_CTX-M_ gene used the primers CTX-Fwd and CTX-Rvs (Dierikx et al., 2012), which amplified a 593 bp subfragment of the 876 bp *bla*_CTX-M_ gene. An 846 bp subfragment of IS*Ecp1* was amplified using IS*Ecp1*-Fwd and IS*Ecp1*-Rvs (This study). PCR for *gfp* in the *E. coli* recipient strain CV601 used the primers P_gfp_ (up) and P_gfp_ (down), (Andersen et al., 1998), which amplified a 714 bp subfragment of the *gfp* gene. All PCR products were electrophoresed and visualised on a 1% TAE agarose gel run at 85 V for 1.5 h using a 100 bp Quick-Load® DNA Ladder (NEB, USA) as a marker and visualised using a Bio-Rad Universal Hood II-GelDoc System (Bio-Rad, USA).

**Table 1:** Primer sets utilised within PCR analyses with gene specific T_A_ and extension times at 72 °C given in seconds (secs) or minute(s) (min(s)).

Primer name	Direction	Sequence 5′-3′	Product size	T_A_ and time	Extension time at 72 °C	Ref. (if applicable)
CTX-Fwd	Forward	ATGTGCAGYACCAGTAARGTKATGGC	596 bp	55 °C for 30 s	1 min	Dierikx et al. (2012)
CTX-Rvs	Reverse	TGGGTRAARTARGTSACCAGAAYSAGCGG				
*ISEcp1*-Fwd	Forward	CTCTGCGGTCACTTCATTGG	846 bp	54 °C for 1 min	2 min	Designed for this study
*ISEcp1*-Rvs	Reverse	CACCGCCATGTCGTATTTGG				
*P_gfp_* (up)	Forward	ATATAGCATGCGTAAAGGAGAAGAACTTTTCA	714 bp	54 °C 2 min	1 min	Andersen et al. (1998)
*P_gfp_* (down)	Reverse	CTCTCAAGCTTATTTGTATAGTTCATCCATGC				

### 2.5 Sanger sequencing, whole genome sequencing (WGS), assembly, and annotation

Sanger sequencing of PCR products was performed by Eurofins Genomics (Wolverhampton, UK). PacBio long-read WGS of strain 774 was conducted by the University of Liverpool, UK (Liverpool Genomics) using 10 kb libraries with 120 times coverage. Illumina short-read WGS and MinION long-read sequencing of ESBL-Ec isolates and transconjugants was carried out at the University of Cardiff, UK. Illumina sequencing library preparation was conducted using the Nextera XT v2 kit (Illumina, Cambridge, UK) with bead-based normalisation for library quantification measurements. Libraries were sequenced using the Illumina MiSeq using a v3 600-cycle kit (Illumina). The read length was 2×300 bp, before trimming. The gDNA for MinION sequencing was first subject to high-performance isolation and purification via Solid Phase Reversible Immobilisation (SPRI) bead clean up (Beckman-Coulter, High Wycombe, UK). Library preparation for MinION sequencing was conducted using the SQK-RBK110.96 rapid barcoding kit according to the manufacturer’s instructions (Oxford Nanopore Technologies (ONT), Oxford, UK). Sequencing was conducted on R9.4 flow cells (ONT, Oxford, UK). The rapid barcoding library kit generated read lengths of between 200 bp and 60 kb. Following quality trimming with Trimgalore (v0.6.4) and Filtlong (v0.2.1) for Illumina and ONT data, respectively, fastq raw sequences were hybrid assembled using Unicycler (v0.4.7) (Wick et al., 2017). Bioinformatic sequence analysis was completed using Geneious Prime (Dotmatics, Boston, USA), NCBI (National Institutes of Health, Maryland, USA), Snapgene (GSL Biotech LLC, Boston, USA) (all using standard parameters), and programmes available from the Centre for Genomic Epidemiology (CGE) set at standard parameters, including MLST 2.0 (Larsen et al., 2012), Res Finder 4.1 (Bortolaia et al., 2020), and Plasmid Finder 2.1 (Carattoli et al., 2014). The nucleotide sequence for IS*Ecp1* (accession number AJ242809) was downloaded from ISfinder, and a custom database was created within ABRicate (v1.0.0) to screen for the presence (and identify location) of the insertion sequence in all genomes. Whole genome sequences are deposited in NCBI under Bioproject number PRJNA1196928 with the following Accession numbers: SAMN45706859-SAMN45706910.

### 2.6 Core-genome phylogenetics and SNP distance comparison

A core genome phylogeny and SNP distance comparison were conducted on 37 of the 39 *bla*_CTX_ isolates (with isolates EcoHS11212-878 (878) and EcoHS11212-880 (880) removed during quality filtering due to poor sequencing coverage and assembly) and 105 ST2325 genomes downloaded from Enterobase (downloaded April 2022 with available metadata on location and source recorded). Isolate EcoMHE1212-939 (939) with the best sequencing coverage and assembly was used as the representative reference genome for SNP-based data generation, with variant calling performed using Snippy (v4.6.0), using default parameters. Recombination sites were removed with Gubbins (v2.3.4), resulting in 13,143 SNPs in conserved genomic regions. The reference genome was annotated using Bakta (v1.9.3) and bedtools (v2.31.1) and was used to classify SNPs either within gene regions (*n* = 12,343 SNPs) or in intergenic regions (*n* = 826). Snp sites (v2.5.1) were used to extract SNP positions, and IQ-tree (v2.0) was used to generate a phylogeny. SNP pairwise distances were generated using snp-dists (v0.6). The phylogenetic tree was mid-rooted and annotated using iTOL v5.7 (Letunic and Bork, 2021).

### 2.7 Transposition experiments

Four transposition experiments were performed by adapting the methods of Lartigue et al. (2006) and Nordmann et al. (2008). To define the baseline frequency of transfer, transposition of IS*Ecp1 bla*_CTX-M-15_ into endogenous plasmids and transfer of the plasmids into the *E. coli* K-12 CV601 recipient strain were performed in a non-selective (n/s) environment of LB broth with no added antibiotics. To determine if the presence of sub-lethal levels of the antibiotics could enhance the transfer frequency above the baseline transposition/transfer conditions, the experiments detailed above were performed in the presence of the third-generation cephalosporin ceftazidime (CAZ) and the penicillins ampicillin (AMP) and cloxacillin (CLOX), with the sub-lethal levels of antibiotic concentrations tested listed in Table 2. These antibiotics were chosen as they are commonly used in dairy farms globally, with CLOX often favoured for use in dry cow therapy (González Pereyra et al., 2015; Johnson et al., 2016; Breser et al., 2018; Liu et al., 2018; Whitfield and Laven, 2018; Rossi et al., 2019; McDougall et al., 2021). The third-generation cephalosporin (CAZ) was also chosen to act as a positive control for enhanced transposition, as previous studies by Lartigue et al. (2006) and Nordmann et al. (2008) demonstrated enhanced transposition of IS*Ecp1* in the presence of this antibiotic.

**Table 2:** Transposition antibiotics and concentrations tested.

Antibiotic	Abbreviation	MIC	1/2 MIC	1/4 MIC	1/10 MIC
Ampicillin	AMP	32 μg ml^−1^	16 μg ml^−1^	8 μg ml^−1^	4 μg ml^−1^
Ceftazidime	CAZ	1 μg ml^−1^	0.5 μg ml^−1^	0.25 μg ml^−1^	0.1 μg ml^−1^
Cloxacillin	CLOX	256 μg ml^−1^	64 μg ml^−1^	32 μg ml^−1^	25.6 μg ml^−1^

MIC breakpoints were utilised from CLSI (CLSI, 2022) for establishing antibiotic concentrations for enhanced transposition.

From an 18 to 20 h culture grown on non-selective LB agar at 37°C, a single colony suspension of the donors EcoSL1010-687 (687), EcoHS11212-876 (876), EcoMHE1801-956 (956), and EcoSS2501-961 (961) was made in 5 mL LB broth with and without added sub-inhibitory levels of antibiotics as listed in Table 2. A single colony suspension of the *E. coli* K-12 recipient CV601 (*gfp*, Kan^R^, Rif^R^) (Smalla et al., 2000) was also prepared in 5 mL LB broth containing 50 μg ml^−1^ of KAN and grown for 18 h at 37°C shaking at 180 RPM. Donor cultures were diluted 1 in 100 into 5 mL LB broth and grown for 3 h at 37°C, shaking at 120 RPM. The *E. coli* CV601 culture was centrifuged at 8,000 *g* for 5 min in 1 mL aliquots and the pellet washed twice in 1 mL of sterile Maximum Recovery Diluent (MRD; Sigma-Aldrich, USA), with a final resuspension in 1 mL MRD. Cultures were diluted using MRD to approximately 0.5–0.7 OD_600_ to achieve a culture containing ~1×10^8^ CFU ml^−1^. Conjugation was performed using a 1:4 ratio of donor to recipient culture in a total volume of 1 mL. The conjugation mix was gently vortexed and then incubated at 37°C for 3 h without agitation. Mating was stopped by vigorous vortexing of the conjugation mix culture and placing the culture on ice. A 10^0^–10^−7^ serial dilution in MRD was plated in duplicate onto LB agar containing 100 μg ml^−1^ AMP (according to the method of Nordmann et al., 2008; IS*Ecp1* selective marker) and 50 μg ml^−1^ KAN (CV601 selective marker) in 0.1 mL volumes. Plate counts for each donor and the recipient were also conducted by plating 100 μL in duplicate of the 10^0^–10^−7^ serial dilutions in MRD, onto LB plus 100 μg ml^−1^ AMP for the donors and LB plus 50 μg ml^−1^ KAN for CV601. All plates were incubated for 18 h at 37 °C. Colonies were counted and CFU ml^−1^ calculations conducted on the following day.

Successful transconjugants were designated ‘transposition transconjugants’ (TT) as there had been transposition of IS*Ecp1* into an endogenous plasmid, followed by conjugation of the IS*Ecp1*-containing plasmid to the recipient *E. coli* K-12 CV601 strain. Presumptive TT colonies from LB AMP100 KAN50 plates were selected for further confirmation by looking for expression of recipient GFP, detected if they fluoresced when exposed to UV illumination at a wavelength of 365 nm, using a UVGL58 UVP Dual Tube Handheld UV Lamp (ThermoFisher Scientific, Loughborough, UK). Colonies that fluoresced were then counted, and the CFU/ml^−1^ calculated. The transposition frequency was calculated as the ratio of CFU/ml of transconjugants to CFU/ml of donors. The plasmids contained within the donor strains were all cryptic, with no selectable genetic markers, so it was not possible to obtain a conjugation frequency for each plasmid alone. Therefore, the final transfer frequency was a combination of the transposition and conjugation frequencies. Transfer frequencies were calculated from counting fluorescent GFP-positive colonies. Colonies confirmed as a successful TT were restreaked onto double selective media purification plates, containing both AMP100 and KAN50 to create a pure culture, and these were banked as the confirmed TT strains for further study.

DNA was extracted using the simple boiling method (Wei, 2013) from single TT colonies from each of the purification plates. To ensure isolates were true transconjugants, rather than mutated donors, confirmation was achieved through PCR for *bla*_CTX-M_, IS*Ecp1,* and *gfp* to show successful mobilisation of IS*Ecp1* in association with *bla*_CTX-M_ and transfer into the recipient CV601 strain.

### 2.8 IS*Ecp1* plasmid typing and insert location

A total of 15 TTs were sequenced using Illumina short read and MinION long read WGS to further analyse the plasmid types that had transferred into the CV601 recipient. The genes located between the IS*Ecp1* IRs were also investigated to understand which genes had been transferred or lost during transposition. The sequences were first analysed through CGE online software^1^ using PlasmidFinder 2.1 (Joensen et al., 2014) to identify contigs containing plasmid replicons. Further sequence analysis was conducted in Geneious Prime, 2023 (version 2023.2.1),^2^ which included assessing the size of the new IS*Ecp1* genetic environments within the TT background, construction of the various IS*Ecp1* genetic environments and plasmid backbones, and searching the surrounding chromosome of the TTs.

IS*Ecp1* element sizes were assessed through the identification of first the IR_L_, and then the identification of the 5 bp repeats and the new IR_R_. The sequences of the TTs are available under Bioproject number PRJNA1196928; a full list of accession numbers is listed in Supplementary Table S4.

## 3 Results

### 3.1 MIC data for selected strains confirmed previously reported resistance

MIC analysis on the 39 strains for seven of the antibiotics confirmed previously reported phenotypic resistance data (Baker et al., 2022) for ampicillin (AMP), ceftazidime (CAZ), cefotaxime (CTX), cefpodoxime (CPD), cefquinome (CFQ), aztreonam (ATM), and tetracycline (TET). The full list of resistant MIC results is available in Supplementary Table S5.

All 39 strains displayed high-level resistance to AMP, CTX, CPD, and CFQ, consistent with *bla*_CTX-M-15_ carriage. Additionally, all strains were resistant to CAZ, but the MIC was at a much lower value of 16 μg ml^−1^ compared to the other third-generation cephalosporins, which had MICs of >512 μg ml^−1^ for CTX and 512 μg ml^−1^ for CPD. The MICs for CFQ and ATM were 128 μg ml^−1^ and 32 μg ml^-1,^ respectively. EcoMHE1801-950 (950), EcoMHE1801-953 (953), EcoMHE1801-955 (955), and EcoMHE1801-956 (956) were the only isolates that were susceptible to TET, with an MIC of <2 μg ml^−1^. The remaining strains were TET resistant, which included seven isolates with an MIC of 128 μg ml^−1^ and the rest with an MIC of 64 μg ml^−1^. MIC results for all the strains for the remaining 19 antibiotics (Supplementary Table S6) tested indicated that they were sensitive.

### 3.2 All selected isolates contained *bla*_CTX-M_ and IS*Ecp1*

The 39 isolates selected for their ESBL phenotype were resistant to the β-lactam AMP, the third-generation cephalosporins CAZ, CTX, CPD, and the monobactam ATM (Baker et al., 2022). This confirmed AST testing data that suggested that the likely resistance mechanism was a *bla*_CTX-M_ type ESBL. PacBio sequencing of ESBL isolate 774, (Baker et al., 2022) had already identified *bla*_CTX-M-15_ in the chromosome, in association with IS*Ecp1*. PCR screening and Sanger sequencing of the remaining 38 strains (data not shown) confirmed they all carried both *bla*_CTX-M_ and IS*Ecp1*. CTX-M typing of the Sanger sequenced PCR products using NCBI BLAST searches, typed all 38 strains as *bla*_CTX-M-15_, which was confirmed as a single copy on the chromosome using WGS.

### 3.3 Sequence mapping demonstrates clonality of the ESBL-Ec isolates

**Figure 1:**
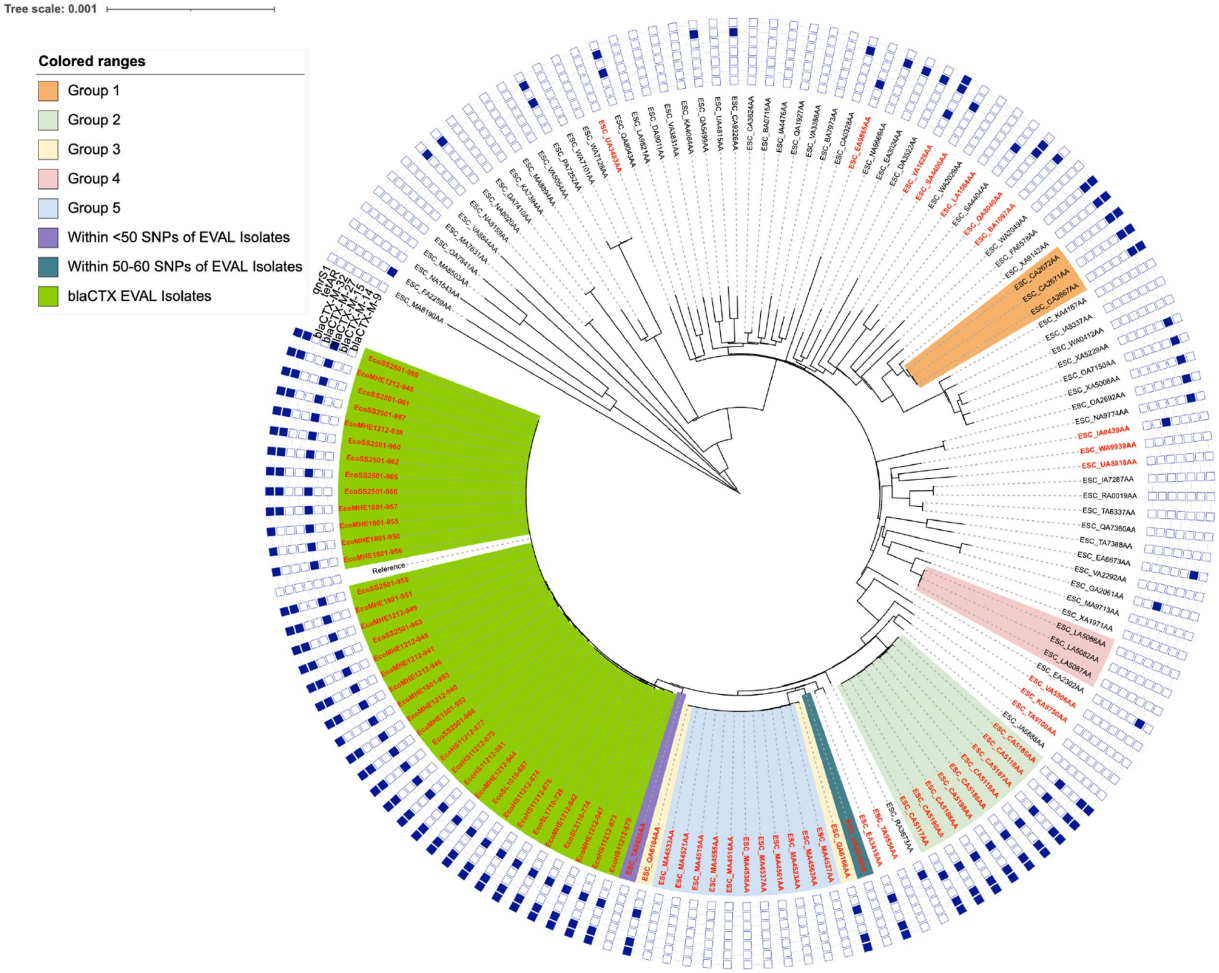
The whole genome phylogeny maximum likelihood tree generated using IQtree v2.0 with annotation achieved using the iTOL v.5.7, showing the 37 *bla*_CTX_ isolates in combination with the 105 ST2325 genomes downloaded from Enterobase. The resistance gene carriage of each isolate is annotated around the outside of the tree, corresponding to the resistance genes *qnrS1*, *bla*_CTX-M-32_, *bla*_CTX-M-27_, *bla*_CTX-M-15_, *bla*_CTX-M-14_ and *bla*_CTX-M-9_, with positive carriage denoted as a filled blue square. Any isolates positive for IS*Ecp1* had the tree label shown in red and the 37 *bla*_CTX_ isolates were highlighted in green. The colour range key and shaded clades on the tree, relates to groups of isolates that were identified as possible clonal groups from the SNP distance comparison.

Multi-locus sequence typing (MLST) of the WGS of the 39 *bla*_CTX_ isolates determined that all were sequence type (ST) 2,325, indicating they could be closely related. ST2325 carrying ESBL has been found in livestock and animal produce; ducks (Yu et al., 2021), farmed rabbits (Silva et al., 2024), raw milk (Irrgang et al., 2017), pigs (Ding et al., 2021), and sheep (Atlaw et al., 2021), as well as in wildlife; gulls (Nesporova et al., 2024). ST2325 has also been found in humans and sewage (Zahra et al., 2018; Ding et al., 2021). To support the idea that ST2325 may form clonal clusters within other animal groups or in bovine-associated studies, a further 105 ST2325 isolates were downloaded from Enterobase (termed Enterobase isolates) for broader SNP distance comparison and phylogenetic analysis. The pairwise SNP distance comparison consisted of the 37 *bla*_CTX_ isolates and the 1 reference genome: isolate 939 (the two isolates 878 and 880 were removed during quality filtering due to poor sequencing coverage and assembly, and isolate 939 was included as both an isolate and as a reference, so was counted twice, resulting in a total of 37 isolates plus the reference). This SNP distance comparison revealed that the 37 *bla*_CTX_ isolates were within 0–6 pairwise SNPs, suggesting a clonal relationship (Figure 1). A few groups of ST2325 Enterobase isolates also formed genetic clusters on the phylogeny, suggesting the presence of several lineages (labelled Group 1–5, Figure 1). Only one ST2325 Enterobase isolate was close in similarity to this study’s isolate cohort, and this was a Spanish bovine isolate that was within <50 SNPs (coloured purple on the tree in Figure 1).

Within the clonal groups from Enterobase, Group 1 was within 0–2 pairwise SNPs, Group 2 was within 0–6 pairwise SNPs, Group 3 was within 0–2 pairwise SNPs, Group 4 was within 0–2 pairwise SNPs, and Group 5 was within 0–4 pairwise SNPs. The *bla*_CTX_ dairy farm isolates and each group alone appeared to be clonal but unrelated to any other groups or isolates. Only Groups 3 and 5 appeared to be clonally related to each other, as all isolates were within 1–4 SNPs of each other and they appeared on the same clade (Figure 1). No other groups appeared to be closely related to each other, the closest being the combined Group 3 and 5, which were within 58–72 SNPs of the *bla*_CTX_ EVAL farms isolates. The two separate isolates ESC_TA9425AA (denoted as <50 SNPs of the dairy farm isolates on Figure 1) and ESC_UA8616AA (denoted as 50–60 SNPs of EVAL isolates on Figure 1) were within 36–45 and 49–58 SNPs of the *bla*_CTX_ dairy farm isolates, respectively, and therefore were the most closely related of the Enterobase isolates to the *bla*_CTX_ EVAL farms isolates. The SNP distance analysis clearly showed, however, that there were multiple sets of evidence for clustering of ST2325 isolates, but at the same time, there was some genomic diversity, which could suggest the international spread and adaptation of a successful clonal lineage. Full details of each group from the tree, the SNP information, full metadata for each isolate, including sampling geographical location and niche, and accession number are available in Supplementary Table S7. A complementary ANI approach provided similar genomic insights into the ST2325 EVAL and Enterobase collections, and details are available in Supplementary Note S2.

### 3.4 *bla*_CTX_ resistance/mobile genetic determinants in the ST2325 collection

Of the 105 ST2325 Enterobase isolates, a total of 38 were found to be encoding IS*Ecp1,* with 17 also encoding a *bla*_CTX-M_. All but 2 of the 17 were of *bla*_CTX-M-15_ type, with the remaining two of *bla*_CTX-M-32_ and *bla*_CTX-M-27_ type. The assemblies of the Enterobase genomes were largely derived from short-read-only sequencing data (which is indicative of limited quality for genomic context analyses), and therefore, it was difficult to be certain whether the IS*Ecp1* genetic environments were located chromosomally or in a plasmid. Manual inspection of the genome in all isolates positive for both IS*Ecp1* and *bla*_CTX-M_ was conducted to confirm where IS*Ecp1* and *bla*_CTX-M_ were in relation to one another. From this, IS*Ecp1* was in the same region as *bla*_CTX-M_ in 16 of the 17 IS*Ecp1* and *bla*_CTX-M_ positive Enterobase isolates, as the IS*Ecp1* transposase was located directly upstream of the *bla*_CTX-M_ gene in the same contig. In the isolate encoding both IS*Ecp1* and *bla*_CTX-M-32_, the *bla*_CTX-M-32_ was found in the middle of a large contig surrounded by what appeared to be chromosomal DNA, which was different from the contig containing IS*Ecp1*.

In addition, 13 of the 15 IS*Ecp1* and *bla*_CTX-M-15_ positive Enterobase isolates were found to be encoding both *tetAR* and *qnrS1,* with the remaining 2 of the 15 encoding either *qnrS1* or *tetAR* alone. The IS*Ecp1* was located in the same region as *tetAR* in 2 isolates and in the same region as *qnrS1* in 4 isolates. In only 1 isolate did both *tetAR* and *qnrS1* appear to be located in the same region as IS*Ecp1*. However, as stated above, due to the fragmented assemblies of the Enterobase genomes, it was unclear which contigs made up the entirety of the IS*Ecp1* genetic environment. Therefore, the relative locations of the *tetAR*, *qnrS1,* and IS*Ecp1* genes were merely an observation from the available data. The association of IS*Ecp1* with ST2325 showed that IS*Ecp1* was quite widespread throughout the 105 isolates, being found in 37.1%. In addition, the most commonly found *bla*_CTX-M_ variant in association with IS*Ecp1* was *bla*_CTX-M-15_, which was the same as the *bla*_CTX_ isolates in this study This could suggest that ST2325 may have an association with IS*Ecp1* and *bla*_CTX-M-15_ but considering the sample size available from Enterobase was small, it is difficult to be certain how widespread IS*Ecp1* and *bla*_CTX-M-15_ are throughout ST2325 isolates that are not represented in the database.

The highest represented niche was livestock, which made up 60% (64 isolates) of the Enterobase isolates examined. Within the livestock niche, the highest numbers were bovine samples, 31.4% (33 isolates), followed by poultry/avian 14.3% (15 isolates) and ovine/goat 9.5% (10 isolates). This could suggest that there is an association between ST2325 and bovine; however, bovine and poultry are more intensively farmed than sheep or goats (Pandey and Upadhyay, 2022) and therefore, the information available for those groups within the repositories may be limited compared to other higher-represented groups such as bovine and poultry. However, it is difficult to determine this, with the limited amount of data available to download for each animal group.

### 3.5 Whole genome sequencing of the 39 isolates identifies five plasmid replicon types and chromosomal carriage of all ARGs and IS*Ecp1* elements

Hybrid assembly of the WGS of the 39 isolates resulted in mostly complete chromosomes and plasmids, allowing high confidence in identifying where resistance genes were located in the genome and good accuracy for estimating plasmid sizes. Supplementary Table S8 shows the full assembly statistics and also includes the ORF number, contig number, contig numbers containing a plasmid, whether the plasmids were complete, overall genome size, overall %GC content, N50 number, and whether the chromosome was complete.

ResFinder results and manual searches of the WGS identified, in addition to *bla*_CTX-M-15_, the resistance genes: *qnrS1,* which provides low-level quinolone resistance (Allou et al., 2009) in all isolates, and *tetAR*, responsible for tetracycline resistance, in all but isolates 950, 953, 955, and 956. These latter isolates had shown susceptibility to tetracycline during AST disc and MIC assays. PlasmidFinder analysis showed that at least 5 plasmid replicon types were present in the isolates, including: IncFIC, IncFII, IncI1, IncI2, and IncX4. In addition to strain 774, two further isolates had no plasmids, and one isolate contained only a single plasmid. All of the plasmids lacked antibiotic resistance genes. Supplementary Table S8 gives full details of isolate plasmid carriage, replicon type, and the contig it was located in.

### 3.6 IS*Ecp1* carrying *bla*_CTX-M-15_ can transpose from environmental isolates into recipient strains

IS*Ecp1 bla*_CTX-M-15_ transposition/conjugation experiments were undertaken using four representative isolates, 687, 876, 956, and 961, and the CV601 *E. coli* K-12 recipient strain. These donor strains carried endogenous plasmid replicon types IncFIC, IncFII, IncI1, IncI2, and IncX4 (Supplementary Table S8 details which plasmid replicon types were within each isolate). The method utilised the endogenous plasmids as vectors and included examples of all the plasmid replicon types observed in the 39 strains.

Of the 40 combinations of 4 donor strains and antibiotic-supplemented growth conditions used for transposition experiments (Table 3), under our experimental conditions, 23 resulted in the isolation of transconjugants. KAN- and AMP-resistant putative transposition transconjugant (TT) colonies that fluoresced under UV light were tested by PCR for the presence of *bla*_CTX-M_ and IS*Ecp1* and for the *E. coli* K-12 recipient CV601 strain *gfp* gene. This confirmed that both *bla*_CTX-M_ and IS*Ecp1* had successfully transferred into CV601 (data not shown).

**Table 3:** Transfer frequencies of IS*Ecp1* element under selective and non-selective growth conditions.

Isolate	Antibiotic	MIC	Concentration	Transfer frequency	Increase from baseline	New TT name	WGS
EcoSL1010-687	Non-selective	n/a	n/a	6 × 10^−8^	–	687-N	Yes
EcoHS11212-876	Non-selective	n/a	n/a	5.03 × 10^−8^		876-N	No
EcoMHE1801-956	Non-selective	n/a	n/a	4.84 × 10^−8^	–	956-N	Yes
EcoSS2501-961	Non-selective	n/a	n/a	3.42 × 10^−8^		961-N	No
EcoSL1010-687	Ampicillin	1/10	3.2 μg ml^−1^	1.79 × 10^−7^	2.98 fold	687AMP0.32	Yes
		1/4	8 μg ml^−1^	1.43 × 10^−7^	2.38 fold	687AMP8	Yes
		1/2	16 μg ml^−1^	4.92 × 10^−8^	No increase	687AMP16	No
EcoHS11212-876	Ampicillin	1/10	3.2 μg ml^−1^	No transfer	–		
		1/4	8 μg ml^−1^	3.33 × 10^−8^	1.67 fold	876AMP8	Yes
		1/2	16 μg ml^−1^	No transfer	–		
EcoMHE1801-956	Ampicillin	1/10	3.2 μg ml^−1^	No transfer	–		
		1/4	8 μg ml^−1^	1.89 × 10^−8^	No increase	956AMP8	Yes
		1/2	16 μg ml^−1^	1.28 × 10^−8^	No increase	956AMP16	Yes
EcoSS2501-961	Ampicillin	1/10	3.2 μg ml^−1^	No transfer	–		
		1/4	8 μg ml^−1^	No transfer	–		
		1/2	16 μg ml^−1^	No transfer	–		
EcoSL1010-687	Cloxacillin	1/10	25.6 μg ml^−1^	No transfer	–		
		1/4	64 μg ml^−1^	7.25 × 10^−8^	1.2 fold	687CLOX64	Yes
		1/2	128 μg ml^−1^	6.67 × 10^−8^	1.1 fold	687CLOX128	Yes
EcoHS11212-876	Cloxacillin	1/10	25.6 μg ml^−1^	No transfer	–		
		1/4	64 μg ml^−1^	1.15 × 10^−8^	No increase	876CLOX64	No
		1/2	128 μg ml^−1^	2.42 × 10^−7^	1.21 fold	876CLOX128	No
EcoMHE1801-956	Cloxacillin	1/10	25.6 μg ml^−1^	1.90 × 10^−7^	3.93 fold	956CLOX25.6	Yes
		1/4	64 μg ml^−1^	5.19 × 10^−8^	1.07 fold	956CLOX64	No
		1/2	128 μg ml^−1^	1.85 × 10^−8^	No increase	956CLOX128	Yes
EcoSS2501-961	Cloxacillin	1/10	25.6 μg ml^−1^	1.08 × 10^−8^	No increase	961CLOX25.6	Yes
		1/4	64 μg ml^−1^	1.25 × 10^−8^	No increase	961CLOX64	Yes
		1/2	128 μg ml^−1^	No transfer	–		
EcoSL1010-687	Ceftazidime	1/10	0.1 μg ml^−1^	2.70 × 10^−6^	45 Fold	687CAZ0.1	No
		1/4	0.25 μg ml^−1^	1.25 × 10^−6^	21 Fold	687CAZ0.25	No
		1/2	0.5 μg ml^−1^	6.98 × 10^−8^	1.16 Fold	687CAZ0.5	No
EcoHS11212-876	Ceftazidime	1/10	0.1 μg ml^−1^	No transfer	–		
		1/4	0.25 μg ml^−1^	2.00 × 10^−8^	1 fold	876CAZ0.25	Yes
		1/2	0.5 μg ml^−1^	No transfer	–		
EcoMHE1801-956	Ceftazidime	1/10	0.1 μg ml^−1^	No transfer	–		
		1/4	0.25 μg ml^−1^	6.52 × 10^−8^	1.34 fold	956CAZ0.25	No
		1/2	0.5 μg ml^−1^	5.66 × 10^−8^	2.83 fold	956CAZ0.5	No
EcoSS2501-961	Ceftazidime	1/10	25.6 μg ml^−1^	No transfer	–		
		1/4	64 μg ml^−1^	No transfer	–		
		1/2	128 μg ml^−1^	No transfer	–		

Baseline transfer frequencies at the top of the table are shaded in green. n/a, not applicable; NG, no growth; TMTC, too many to count.

### 3.7 Sublethal concentrations of antibiotics can lead to an enhanced transfer frequency of IS*Ecp1*

The transfer frequency of the IS*Ecp1* element represents a combination of the initial transposition of IS*Ecp1* from the chromosome into a resident plasmid, followed by conjugation of the plasmid into the *E. coli* K-12 recipient strain CV601. Successful transposition/conjugation of the IS*Ecp1* element occurred in the absence as well as presence of AMP, CLOX, and CAZ in the LB media in the conjugation assays were carried out in, and at each of the concentrations of antibiotic that were used (Table 3). However, the frequency of transposition/conjugation varied with each donor strain. Furthermore, enhanced frequency of transposition and transfer into the CV601 host was successful with all the concentrations of CLOX and CAZ tested, but only with two concentrations of AMP (0.32 and 8 μg ml^−1^). The transfer frequency for each donor with each antibiotic and the increase from the baseline frequency of transfer and new TT names are also shown in Table 3.

The baseline transfer frequency of IS*Ecp1* from each donor to the recipient CV601, when isolates were grown in nonselective medium with a starting culture of 1 × 10^8^ cells, was 6 × 10^−8^ with isolate 687 (around 1 in 16 million), 5.03 × 10^−8^ with isolate 876 (around 1 in 19 million), 4.84 × 10^−8^ with isolate 956 (around 1 in 20 million) and 3.42 × 10^−8^ with isolate 961 (around 1 in 29 million), which are lower than with artificially constructed donors (Lartigue et al., 2006; Nordmann et al., 2008). An enhanced frequency of transfer above the baseline frequency was only observed in 687, 876, and 956, and with only some of the antibiotics and concentrations tested.

The majority of enhanced transposition/ conjugation (transfer) frequencies were seen with 1/10 to 1/4 MIC concentrations of antibiotics, and this was evident for AMP in 687, which produced a slightly higher transfer frequency of 2.98-fold at 1/10 MIC than 2.38-fold at 1/4 MIC. In 687, CLOX had a slightly higher transfer frequency of 1.2 fold at 1/4 MIC than 1.1 fold at 1/2 MIC. In 956, CLOX showed a higher frequency of 3.93 fold at 1/10 MIC than 1.07 fold that was seen at 1/4 MIC. The biggest increase in enhanced transfer was seen with CAZ in 687, where there was a much higher frequency of transfer of 45-fold at 1/10 MIC than 21-fold at 1/4 MIC. In comparison, however, in 956, the enhanced frequency of transfer with CAZ was greater at 2.83 fold at 1/2 MIC than at 1.34 fold at 1/4 MIC. No enhanced transfer frequencies were observed with any antibiotics in 961, and there were no enhanced transfer frequencies with AMP in 876 and 956 or with CAZ in 876.

As shown in Table 3, of the 23 conjugations that show a successful transfer, 15 transconjugants (that represented a combination of each donor strain and each concentration of AMP and CLOX tested) were sequenced using Illumina short read and MinION long read with hybrid assembly.

**Figure 2:**
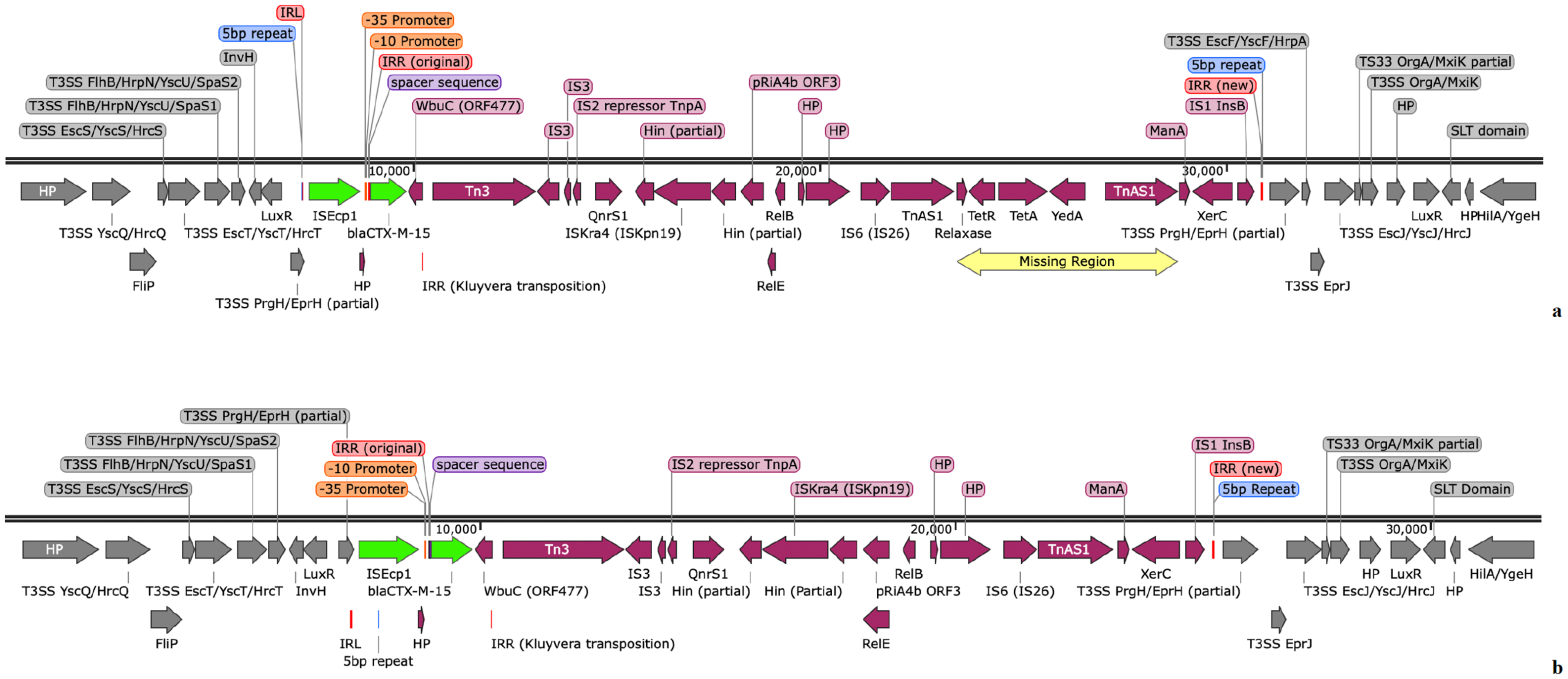
**(A)** IS*Ecp1* chromosomal environment of isolate EcoSL3110-774 from the PacBio sequence (IS*Ecp1* and *bla*_CTX-M-15_ are shown in green with the remaining genetic environment shown in maroon), showing the 5 bp repeats (blue), IR_L_ and IR_R_ (red) and the surrounding chromosome (grey) around the insertion of IS*Ecp1*, with the area denoted as a yellow box and annotated as ‘missing region’, detailing the area missing from within the tetracycline susceptible isolates 950, 953, 955, 956 and 962 shown in **(B)** the smaller IS*Ecp1* element found in the chromosome of isolates 950, 953, 955, 956 and 962 which all were missing the region of TetAR, YedA and TnAS1. The IS*Ecp1* and *bla*_CTX-M-15_ are shown in green with the remaining genetic environment shown here in maroon, the 5 bp repeats are in blue, the IR_L_ and IR_R_ are in red and the surrounding chromosome around the insertion of IS*Ecp1* is in grey.

The CGE MLST programme (Larsen et al., 2012) was used to confirm from the WGS data that all putative transconjugants except isolate 687AMP16 were of ST10, the same as the recipient CV601. Isolate 687AMP16 was found to be the same ST as the parent ST2325; this isolate was therefore discounted from any further analysis. The *gfp* gene was also located successfully within the genome sequence of all the TTs confirmed as being of ST10, further confirming that the TT strains were a result of plasmid transfers into CV601. The IS*Ecp1 bla*_CTX-M-15_ elements were found to have inserted into all but the IncI2 plasmid replicon type plasmids during transfer to the recipient CV601 strain; partial plasmid maps showing the structure of the IS*Ecp1* elements are detailed in Supplementary Note S1 and full details of the insertion points with schematics of the IR_R_ used by each TT and the TT genetic environments are detailed in Supplementary Tables S9, S10 and S11. These data show that in only two of the 13 (~15%) TT examined was the IS*Ecp1* element identical to that of the donor parent: three were larger, having gained additional chromosomal genes from the original host; the rest were smaller, having lost genes from the original IS*Ecp1* structure. Genes gained were from the Type 3 Secretion System (T3SS) located downstream of *bla*_CTX-M-15_ in the donor (Figure 2A). The most significant gene loss was that of the *tetAR* genes in nine TT, with six additionally losing the *qnrS1* gene. These structural changes were all likely a result of the recognition of a new imperfect IR_R_ used by IS*Ecp1* during transposition. From the data on the insertion sites in the plasmids, in some cases, a plasmid conjugation gene was interrupted, which could affect onward transmission of the IS*Ecp1*elements. Thus, although many of the TT would be able to act as conjugation donors for the IS*Ecp1*element with the *bla*_CTX-M_ resistance genes, in only a minority would the *tetAR* and *qnrS1*genes also be transmitted, limiting their spread.

### 3.8 The IS*Ecp1* element provides the same level of phenotypic antibiotic resistance to the recipient strain as was in the donor strain

To determine the effects on phenotypic resistance from the transposition of IS*Ecp1* and transfer of resistance into the recipient CV601 strain, MICs were performed as described for the parent strains on the transposon transconjugants, to assess any changes in the level of resistance. A panel of 15 antibiotics was selected from the original MIC panel of 25 antibiotics (listed in Supplementary Table S2) that included AMP, CAZ, CTX, CPD, CFQ, ATM, AMC, FOX, ertapenem (ERT), neomycin (NEO), TET, nalidixic acid (NAL), ciprofloxacin (CIP), and enrofloxacin (ENR). Additionally, CLOX was included utilising a previously described breakpoint (Hertz et al., 2014).

The recipient strain CV601, which encodes a 3′-phosphotransferase *aph3’,* was only resistant to NEO in the panel of antibiotics, with an MIC of 64 μg ml^-1,^ and was susceptible to all other antibiotics tested. However, following the transposition experiments, the recipient strain was resistant to AMP, CLOX, CAZ, CTX, CPD, CFQ, and ATM, with all TTs showing identical MICs of >512 μg ml^−1^, >512 μg ml^−1^, 16 μg ml^−1^, 512 μg ml^−1^, 128 μg ml^-1,^ and 32 μg ml^−1^, respectively. These results were also identical to the MICs for the donor parent strains. The MICs for AMC, FOX, ERT, and NAL were also identical in all TTs and to the parent donor strains. Only 687CLOX128, 961CLOX64, and 687AMP0.32 were resistant to TET (with an MIC of 64 μg ml^−1^), which was identical to the parent donor strains. All other TTs were susceptible to TET, owing to the loss of the *tetAR* genes or their absence in the parent strains. The only other differences in MICs between the TTs and the parental strains were with CIP and ENR. In 8 of the TTs, which included 687-N, 687AMP0.32, 687CLOX64, 687CLOX128, 956AMP8, 956AMP16, 961CLOX25.6, and 961CLOX64, the MIC results for CIP and ENR were 0.25 μg ml^−1^ and 1 μg ml^-1,^ respectively, which were the same as the parent MIC results. In the remaining TTs, which had lost *qnrS1* during IS*Ecp1* transposition, the MIC for CIP and ENR gave the same MIC result as CV601 of ≤0.064 and ≤0.032, respectively. These MICs demonstrated that the level of phenotypic resistance was maintained following transposition of IS*Ecp1* from the parent donor strains into the recipient CV601, with high-level β-lactam resistance still present.

### 3.9 The IS*Ecp1* elements are fluid, losing or gaining resistance and virulence genes

**Figure 3:**
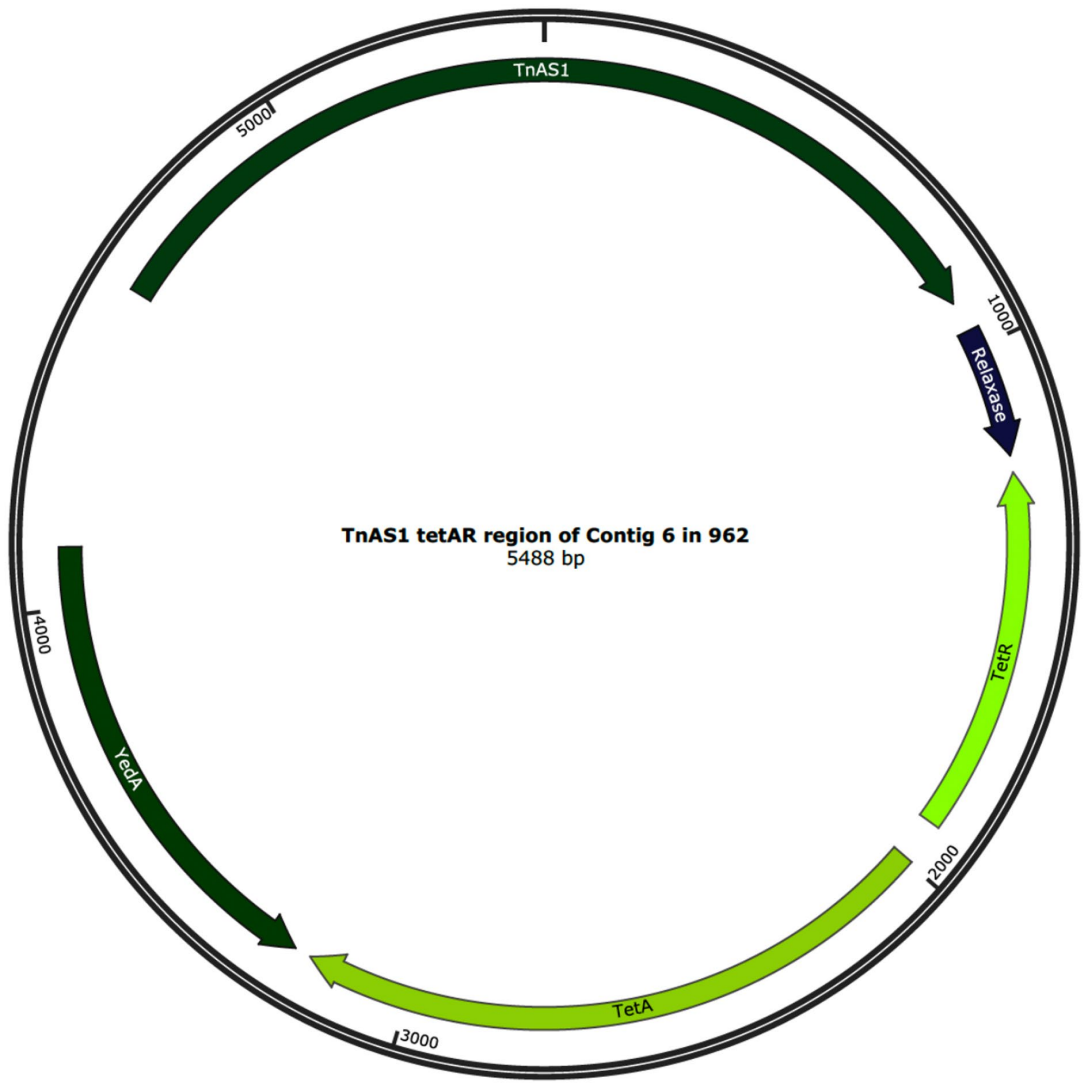
The annotated circularised genomic region of 5,488 bp in contig 6 from isolate 962, showing the complete Tn*AS1* transposase, relaxase, *tetAR* and *yedA.*

The data from the isolate WGS and TTs together demonstrate that the IS*Ecp1* element is both capable of losing and gaining genes during transposition. Thirty-four of the isolates carried IS*Ecp1* elements, which were identical to each other and to that of the principal isolate 774 at 26,612 bp (Figure 2A). However, in isolates 950, 953, 955, 956, and 962, the IS*Ecp1* element was slightly smaller at 18,025 bp (Figure 2B). The smaller size of the IS*Ecp1* element in these five isolates was due to the absence of a 5,587 bp region encoding the tetracycline resistance and a relaxase, respectively *tetAR*, *yedA,* and Tn*AS1*, (denoted as “Missing region” in Figure 2A) which supports the lack of phenotypic tetracycline resistance seen in the isolates 950, 953, 955, and 956. However, isolate 962 did show phenotypic resistance to tetracycline. In isolates 950, 953, 955, and 956, the *tetAR* region was completely absent from the genome; however, in isolate 962, a small, circularised region denoted as Tn*AS1* that encoded *tetAR* was found within a small contig separate from the IS*Ecp1* region, as shown in Figure 3. The 5,488 bp region of the contig in isolate 962 was identical to the Tn*AS1* region of all the other +*tetAR* IS*Ecp1* isolates. As already indicated (Section 3.7), many of the TT showed loss of genes during transposition, including resistance genes.

In addition, analysis of the TTs showed that three of the TTs—687AMP0.32, 687CLOX128, and 961CLOX64—had gained virulence genes from the T3SS by recognising a new imperfect IR_R_ further along the genome during transfer (Section 3.7).

## 4 Discussion

We studied 39 ESBL-Ec isolated from a UK dairy farm to understand how related they were and whether the resistance genes they carried were mobile. We demonstrated that these strains were all ST2325 and within 0–6 SNPs of each other, isolated over 4 months, and from different parts of the dairy farm. WGS of the strains showed that the ESBL phenotype arises from chromosomal carriage of *bla*_CTX-M-15_, located on the mobile genetic element IS*Ecp1,* which was identical in all but five isolates, where a truncated version of the element was present. We showed that the IS*Ecp1* element is fluid, able to gain or lose resistance or virulence genes following transposition; that this element and its resistance genes can be mobilised via plasmids and transfer to recipient strains; that this mobilisation can be enhanced by sublethal concentrations of antibiotics; and that mobilisation leads to acquisition of the resistance phenotype in the recipient strains, at a level that would be of clinical concern. To the best of our knowledge, this is the first time that environmentally occurring resistance genes from environmentally occurring *E. coli* have been shown to transfer using the strains’ own plasmids into a recipient *E. coli* strain, leading to phenotypic resistance. This is important evidence in favour of a One Health approach to AMR (Arnold et al., 2024). These results are also direct evidence for the relevance of the WHO Tricycle protocol for AMR (WHO, 2021), which specifically focusses on surveillance of ESBL-producing *E. coli.* Moreover, our results show that these resistances are dynamic and evolvable. They imply that surveillance must capture those AMR dynamics, whether through longitudinal sampling that can show whether these organisms are increasing or decreasing in prevalence or through sampling of selective agents, such as antibiotics or other chemicals that can select for transfer (Alav and Buckner, 2024), so that the risk of spread of resistance can be evaluated. Equally, a deep understanding of the dynamics of resistance evolution and spread also requires both genome sequencing and laboratory experimentation, which may be beyond the scope of the WHO protocol for some laboratories.

### 4.1 IS*Ecp1*: a vehicle for *bla*_CTX-M_ transmission

This study showed the ability of IS*Ecp1* to mobilise *bla*_CTX-M-15_ both in a nonselective and selective (sub-lethal levels of β-lactam antibiotics) laboratory conditions. Mobilisation of IS*Ecp1* in a non-selective environment suggested that the response to the experimental conditions, such as the ideal physiochemical conditions, high nutrient availability, and a stable temperature, was sufficient for mobilisation of IS*Ecp1* to occur. However, inclusion of sub-lethal levels of the antibiotics CAZ, AMP, and CLOX enhanced transposition of the IS*Ecp1* carrying *bla*_CTX-M-15_ and was likely the result of sub-lethal levels of antibiotics affecting the IS*Ecp1* element as opposed to *bla*_CTX-M-15_. This result was anticipated, as this effect has been previously described (Miller et al., 2004; Aminov, 2011; Beceiro et al., 2013). It is known that sub-lethal levels of β-lactam antibiotics can induce an SOS response, which may lead to increased mutagenic activity and genetic variability with resultant increased MGE mobilisation (Kuan et al., 1991; Capy et al., 2000; Foster, 2007). Other antibiotics, including ciprofloxacin, trimethoprim, and some other quinolones, may induce the SOS response, demonstrating that exposure to one antibiotic could result in the dissemination of resistance to an unrelated antibiotic (Hastings et al., 2004). Studies by Lartigue et al. (2006) and Nordmann et al. (2008) have shown that the presence of CAZ can enhance the transposition of an IS*Ecp1* element. Sub-lethal levels of CAZ enhanced transposition by up to 45-fold, and the other 2 antibiotics used in this study (AMP and CLOX) also enhanced transposition by up to 2.98 and 3.93-fold, respectively. Out of 36 TTs (not including the 4 baseline nonselective controls), 14 showed an enhanced frequency of transfer of IS*Ecp1 bla*_CTX-M-15_ compared to the baseline frequency of transfer, which demonstrated that IS*Ecp1* transposition does not appear to be a rare event. Only a small number of strains were utilised in the enhanced transposition experiments, and many TTs were generated, with some transconjugant plates containing 1 colony from an initial culture plating of only 100 μL, which equates to ~ 1 × 10^8^ CFU/mL. Although the assay we conducted arguably more closely approximates “real world” conditions for transposition into endogenous plasmids and transfer into a new host strain, the transfer rate was a combination of transposition and conjugation, so we cannot exclude other explanations such as enhanced conjugation effects occurring. Møller et al. (2017) demonstrated that high levels of cefotaxime (126 μg ml^−1^) were able to increase conjugation frequencies of an IncI1 plasmid. Following treatment with cefotaxime for 30 min, conjugation frequencies saw an 8.4-fold increase and after 60 min of treatment conjugation frequencies saw a 6.6-fold increase. Møller et al. (2017) also showed through qPCR that certain transfer genes were upregulated in response to cefotaxime treatment. Therefore, it cannot be ruled out that there was some influence on plasmid transfer from the antibiotics used in this study. However, transfer of the IS*Ecp1* elements was evident, as they had originally been chromosomally encoded in the parents and were of varying sizes in the resultant transconjugants, through loss or gain of genes.

### 4.2 IS*Ecp1* as a fluid genetic environment

IS*Ecp1* belongs to the IS*1380* family of IS elements and uses a DDE transposase in a ‘copy-in’ mechanism (Poirel et al., 2005; Bouuaert and Chalmers, 2010). However, unlike other IS elements, it can mobilise downstream genes producing what have been termed ‘transposition units’. IS*Ecp1* mobilises via a one-ended transposition mechanism (Poirel et al., 2005; Kieffer et al., 2020) and is flanked by left and right Inverted Repeats (IR_L_ and IR_R_). A common feature of IS*Ecp1* mobilisation is the recognition of an imperfect IR_R_ (Poirel et al., 2003; Poirel et al., 2005; Lartigue et al., 2006; Dhanji et al., 2011; Zowawi et al., 2015; Sun et al., 2016; Hamamoto et al., 2020; Widyatama et al., 2021) and through this the collection of new genes may occur, into what have been termed transposition units (Zong et al., 2010; Widyatama et al., 2021; Yagi et al., 2021). Due to the recognition of an imperfect IR_R_, these transposition units can vary in size (Poirel et al., 2005). The result is that downstream genes may be collected or lost as mobilisation takes place (Zong et al., 2010), allowing for the capture of adjacent genes further downstream. IS*Ecp1* also provides the −35 and −10 promoter sequences for high level expression of *bla*_CTX-M_ (Poirel et al., 2005; Zong et al., 2010).

The genetic environments of the IS*Ecp1* elements in the transconjugant plasmids varied significantly, and several of the IS*Ecp1* elements had lost both the *tetAR* region and *qnrS1,* whilst retaining *bla_CTX-M-15_*. Loss of these resistance genes showed that IS*Ecp1* transposition can also reduce the resistance gene content of the mobile element, through the recognition of an imperfect IR_R_ within the IS*Ecp1* element, and consequent loss of resistance genes. Recognition of the new imperfect IR_R_ is not random, as there is commonly some homology to the IR_L_, but it does appear to be somewhat random how far downstream from the IR_L_ this recognition happens (Poirel et al., 2008). Therefore, gene gain or loss as a consequence of this degenerate sequence recognition phenomenon is equally possible, and the TTs analysed in this study appear to support this mechanism. Other mobile elements that mobilise by the recognition of variable different IR_R_ sequences are Tn*2* and the insertion sequence IS*91*. The right extremity of the mobile element is defined in both Tn*2* and IS*91* through this mechanism (Poirel et al., 2008).

The finding of a circularised Tn*AS1* (Figure 3) in isolate 962 shows the potential for *tetAR* to mobilise independently from the IS*Ecp1* element and suggests the Tn*AS1* may mobilise via either a copy out and paste in or a cut out and paste in mechanism (Bouuaert and Chalmers, 2010; Skipper et al., 2013).

Clearly, the mobility of the IS*Ecp1* element has implications in antibiotic resistance spread, pathogen evolution, and the silent colonisation of human and animal hosts by antibiotic-resistant *E. coli*. The plasticity of the IS*Ecp1* element extends beyond resistance genes and into the gain of virulence genes, as evidenced by the T3SS genes gained. The T3SS system within *E. coli* is an important factor, critical to virulence in pathogenic *E. coli* strains such as EPEC and EHEC. The T3SS delivers effector proteins to eukaryotic host cells, involved in the subversion of cellular processes, such as signalling pathways within the host, and results in attaching and effacing lesion creation (Ideses et al., 2005; Zhou et al., 2014). A good example of a foodborne pathogen that has a well-defined T3SS is EHEC O157, and healthy cattle are a known reservoir of EHEC O157 (Lim et al., 2010). This demonstrated that through IS*Ecp1* transposition, there is the potential for movement of important virulence genes, which might generate new variants of pathogens, which is a key fundamental biological process.

This acquisition and possible loss of downstream genes may have an impact on the evolution of gene content and, in particular, the “accessory genome” of a bacterium, by introducing or losing genes associated with resistance, virulence, or those genes involved in increased survival and colonisation of niche environments (Medini et al., 2005; Tettelin et al., 2008; Siguier et al., 2014).

### 4.3 Explaining the clonal expansion of the strains in the farm environment

The 39 ESBL *E. coli* isolates taken from the dairy farm over a 4-month period from the slurry tank, the muck heap, and heifer sheds were found to be clonal. This clone was resistant to cephalosporins that had been discontinued on the farm and to those still in use, such as the penicillins. Prior to this study, the last use of first-generation cephalosporins on this dairy farm was in April 2017, the last use of third-generation cephalosporins was in January 2016, and the last use of fourth-generation cephalosporins was in August 2015 (Baker et al., 2022; Todman et al., 2024). However, amoxicillin, benzylpenicillin, cloxacillin, penethamate, and oxytetracycline were used on the farm between 2015-18. The first *bla*_CTX_ *E. coli* strain was isolated from the slurry tank on 10 October 2017 (strain 687), followed by strains isolated on 17 October (762), 31 October (774), 12 December (863–949), 18 January 2018 (950–957), and 25 January (958–96). There had therefore been a period of at least 20 and 24 months between the last use of third and fourth-generation cephalosporins, respectively, and at least 5 months between the last use of first-generation cephalosporins and the isolation of the first *bla*_CTX_ *E. coli* strain, strain 687. This suggests that the use of first-, third-, or fourth-generation cephalosporins was not necessary to maintain this mobile genetic element in *E. coli* strains within the dairy farm, as the isolation of these strains occurred after those antibiotics had ceased to be used there. However, other β-lactam antibiotics were still in use, and because *bla*_CTXM-15_ confers resistance to them, their use may have been exerting a selective effect on carriage of this element.

There are five hypotheses behind the appearance and persistence of this clone: successful colonisation and growth of the ESBL-EC ST2325 strain within the herd, selective isolation media bias in strain recovery, selection by β-lactams, co-selection by tetracycline, or co-selection by copper and zinc ions.

This ESBL-Ec strain (the first isolate of which was strain 687) was first isolated at the end of a continuous period of sampling over nearly 2 years. This is consistent with the idea that the strain may have arisen either by a new strain arriving at the farm and becoming established, or an existing ST2325 strain acquiring the IS*Ecp1* element from another bacterium. It is clear from the sequence analysis that there had been multiple exchanges of plasmids within the different sequenced isolates. The global dissemination of IS*Ecp1* carrying *bla*_CTX-M-15_ has occurred both by clonal expansion of ST131 and by transfer of the element in *Enterobacteriaceae* (Awosile and Agbaje, 2021). That this has been clonal expansion and not repeated transfer of the IS*Ecp1* element is supported by the phylogenetics together with the identical nature of the ISE*cp1* element in farm isolates, which, with the exception of the *tetAR* negative strains, did not demonstrate the high variability of this element evident in TTs.

Whilst we cannot exclude isolation bias, the fact that the ST2325 *E. coli* strain was established for at least 4 months on the farm and was isolated independently on each date amongst many other strains, mitigates against selective isolation bias.

We examined that it is unlikely that environmental β-lactam selection was selecting for IS*Ecp1*, because β-lactams are known to be highly susceptible to hydrolysis. However, some studies appear to show that β-lactams may still be detected within environmental samples. It has been reported that levels of β-lactams are usually at the limit of detection (LOD) within wastewater/effluent or at very low levels, as they are often degraded quickly due to either β-lactamase activity or through their susceptibility to hydrolysis (Zhang and Li, 2011; Kulkarni et al., 2017; Rodriguez-Mozaz et al., 2020). Concentrations reported within effluent have been in the region of LOD – 99.4 ng L^−1^ for AMP (Lin et al., 2008; Rodriguez-Mozaz et al., 2020), 15 ng L^−1^ for CLOX (Watkinson et al., 2007) and LOD, 34 ng L^−1^ and <12 ng L^−1^ for CTX (Gulkowska et al., 2008). Despite hydrolysis, it is also possible that β-lactam breakdown products might still be able to select for resistance (Chio et al., 2023).

Co-selection by tetracycline may be more likely because the IS*Ecp1 bla_CTX-M-15_* element carried the *tetAR* tetracycline resistance genes in most isolates. Tetracycline was used extensively on the farm during 2015–2018, persists in the environment, and high levels of tetracycline resistance were observed (Baker et al., 2022). Finally, modelling of the farm environment predicted that cephalosporin resistance would be expected to be chromosomally encoded and could be driven by co-selection through high concentrations of copper and zinc in the dairy waste from the disposal of metal ion footbaths (Todman et al., 2024). The chromosomal carriage of the IS*Ecp1 bla_CTX-M-15_* element confirms the model prediction of co-selection and provides further evidence for the co-selection hypotheses for clonal expansion.

The information gained from the SNP distance comparison of the ST2325 Enterobase genomes together with the 37 *bla*_CTX_ EVAL farms isolates showed that ST2325 appears to form small clonal groups. This was clear from the genomic data that came from studies reporting clear evidence of clonality in their isolates. Group 1 and 2 (Ludden et al., 2019), Group 3 (no reference available), Group 4 (Boehmer et al., 2018), Group 5 (Atlaw et al., 2021) and was also evident for the isolates of the *bla*_CTX_ group analysed as part of this study. For the majority, the clonality was related to samples from the same studies and geographical area. The exceptions were Groups 3 and 5, which formed a clonal cluster on the tree, were within 1–4 SNPs of each other, and appeared to be from different studies. ST2325 is not a particularly widespread clone within the Enterobase genomes, and, in comparison to a significant dominant clone like ST131, which is represented with >14,000 genomes in Enterobase, the ST2325 numbers were relatively small. However, many of the Enterobase isolates were still only within 300–400 SNPs of each other. As the majority of the ST2325 isolates were identified from animals, with only a few from humans, this could possibly indicate a potential route ST2325 has taken from animals into the human population. However, as was previously stated, a greater number of samples would be required for this hypothesis to be looked at more rigorously.

## 5 Conclusion

Taken together, these results show the highly evolvable nature of IS*Ecp1* elements in environmentally occurring *E. coli* and their importance in the dissemination and transfer of antibiotic resistance. In environmental conditions, they and the *bla*_CTX-M-15_ gene they carry are capable of spreading through clonal expansion of the host strains. They are also capable of both acquiring and losing antibiotic resistance and virulence genes, and of horizontal transfer into recipient bacteria, especially under sublethal concentrations of antibiotics, and thus of mobilisation of clinically relevant resistance. Thus, it is essential to understand not just the prevalence of ESBL-producing *E. coli* as the WHO Tricycle One Health surveillance programme suggests, but also the conditions under which IS*Ecp1* elements can gain and spread resistance, and lose resistance genes, in order to effectively mitigate the global emergency of antimicrobial resistance.

## Data Availability

The datasets presented in this study can be found in online repositories. The names of the repository/repositories and accession number(s) can be found in the article/Supplementary material.

## Footnotes

^1^ www.genomicepidemiology.org

^2^ https://www.geneious.com
