# Supplementary material for: Sublethal concentrations of antibiotics enhance transmission of antibiotic resistance genes in environmental Escherichia coli
Source: Front Microbiol. 2025 Oct 23;16:1675089. doi: 10.3389/fmicb.2025.1675089 (PMC12589106; doi:10.3389/fmicb.2025.1675089)
Supplement: Supplementary file 1 [file Data_Sheet_1.docx]

**SUPPLEMENTARY**

**Supplementary Note 1:**

1. **Partial Plasmids Maps and Insertion Points of IS*Ecp1* following Transconjugation Experiments with CV601**

The IS*Ecp1* *bla*_CTX-M-15_ elements were found to have inserted into all but the IncI2 plasmid replicon type plasmids during transfer to the recipient CV601 strain. Within all but two of the IS*Ecp1* elements, a new imperfect IR_R_ denoted as IR_R(new2)_ was present (which are detailed in supplementary **Tables S9, S10 and S11**), indicative of plasticity of gene content. However, in two of the isolates 956-N and 956CLOX128, the original IR_R(_*_Kluyvera_* _transposition)_ was utilised during transfer, which is shown in the diagram of the IS*Ecp1* element in **Figure 2a.** IR_R(_*_Kluyvera_* _transposition)_ is believed to be the original IR_R_ that was utilised by IS*Ecp1* to capture the ancestral *bla*_CTX-M-1_ type from the progenitor *Kluyvera* spp. chromosome, which was believed to be *K. ascorbata*, and was therefore denoted as IR_R(_*_Kluyvera_* _transposition)_/IR_R(new2)_. Transconjugant plasmid sequencing showed that the IS*Ecp1* elements that had inserted were of varying sizes, with several <10kb in size, which was much smaller than the IS*Ecp1* element found within the parents (see **Figure 2)**. Although the IS*Ecp1* elements were also found inserted at varying locations within each of the plasmid sequences. 8 had inserted in and around the *traI*, *traX* and *finO* region, 7 of which were in IncFII plasmids with 1 in an IncI1plasmid.

The IS*Ecp1* elements that had transposed into the endogenous plasmids were sorted into 3 groups according to their sizes (**Supplementary Table A**). Group 1 were all >15 kb, Group 2 were between 10-15 kb h and Group 3 were <10 kb in length **Supplementary** **Figures 1, 2 and 3** show the IS*Ecp1* elements with only the immediate flanking plasmid backbone region. A full overview of each TT, plasmid replicon type, IS*Ecp1* size, insertion point in the plasmid and IR_R_ sequence utilised by IS*Ecp1*, along with a schematic of the IS*Ecp1* genetic environment for each group can be viewed in Supplementary **Tables S9, S10 and S11**.

| **Supplementary Table A: The 14 TTs grouped on size into Group 1 >15 kb, Group 2 10-15 kb and Group 3 <10 kb** | |
| --- | --- |
| **Group** | **TTs within Group** |
| Group 1 >15 kb | 687CLOX128  961CLOX64  687AMP0.32 |
| Group 2 10-15 kb | 687-N  956AMP8  956AMP16  961CLOX25.6 |
| Group 3 <10 kb | 687AMP8  876AMP8  956-N  956CLOX25.6  876CAZ0.25  687CLOX64  956CLOX128 |

- 1. **Group 1: *ISEcp1* elements >15kb in length inserted into conjugative plasmids**

Group 1 contained the three TTs (687CLOX128, 687AMP0.32 and 961CLOX64) *ISEcp1* elements >15 kb in length and shown in **Supplementary** **Figure 1**. All had gained genes from the original host, likely a result of the recognition of a new imperfect IR_R_ utilised during transposition. The additional genes were from the Type 3 Secretion System (T3SS) located downstream of *bla*_CTX-M-15_ in the donor (**Figure 1**) and encoded *prgH*/*eprH* part of the needle complex, *escF*/*yscF*/*hrpA* a needle major subunit, *eprJ* part of the inner membrane ring, *escJ*/*yscJ*/*hrcJ* part of the inner membrane ring and *orgA*/*mxiK* part of the sorting platform. The only noted interrupted gene as a result of IS*Ecp1* insertion into one of the plasmids was *traC*, in 687CLOX128, encoding an ATPase involved in F-pilus biogenesis, which could result in a reduced conjugation efficiency. No other gene interruptions of note were observed.

| 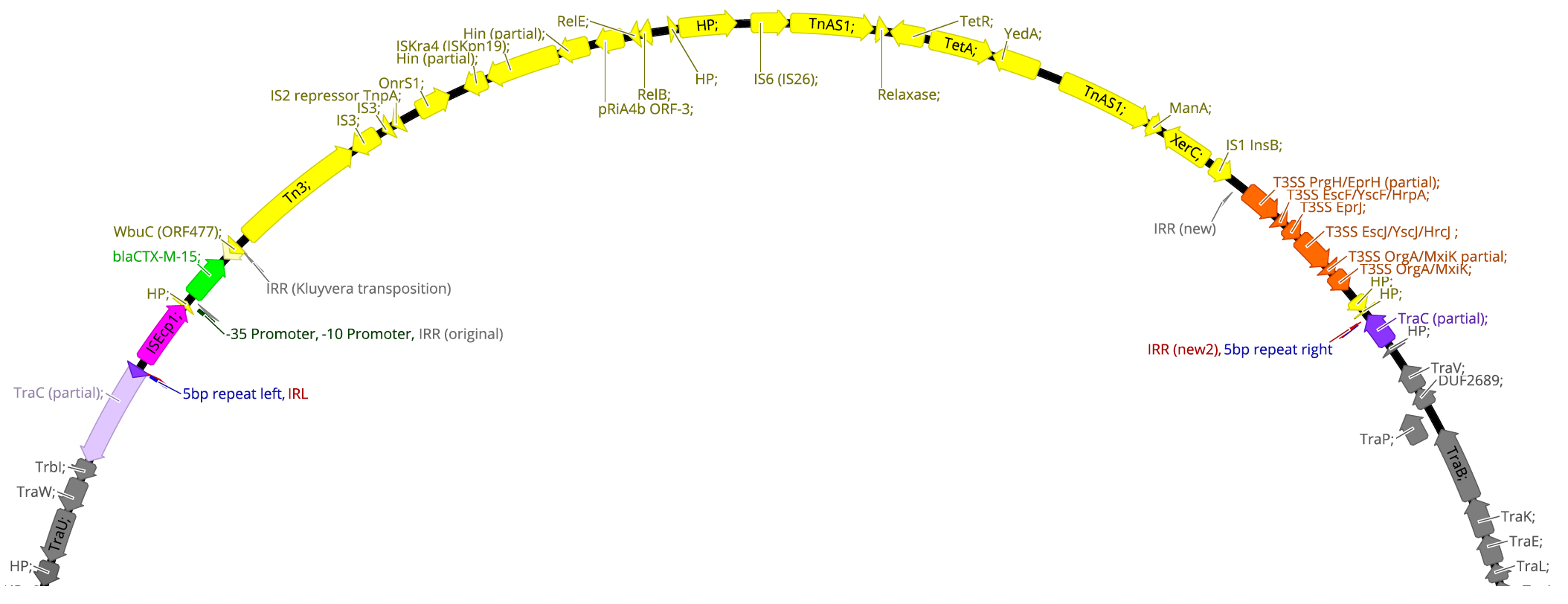 | **a** |
| --- | --- |
| 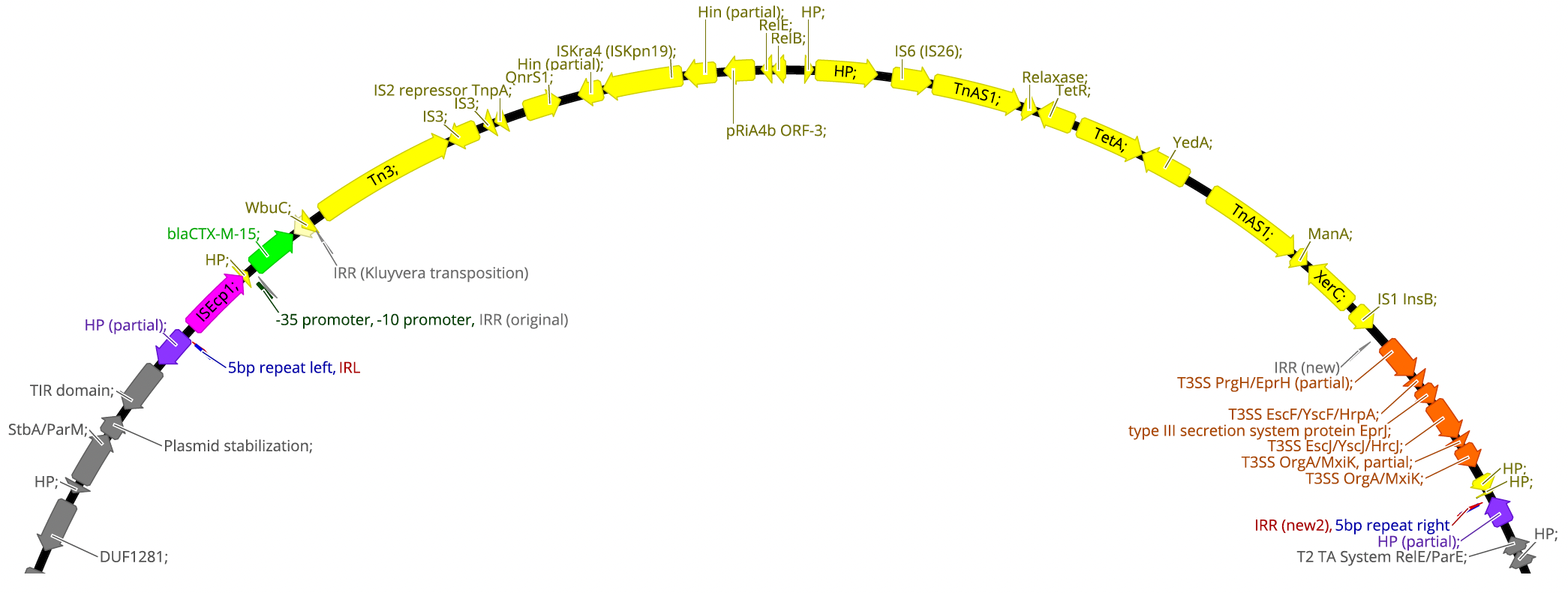 | **b** |
| 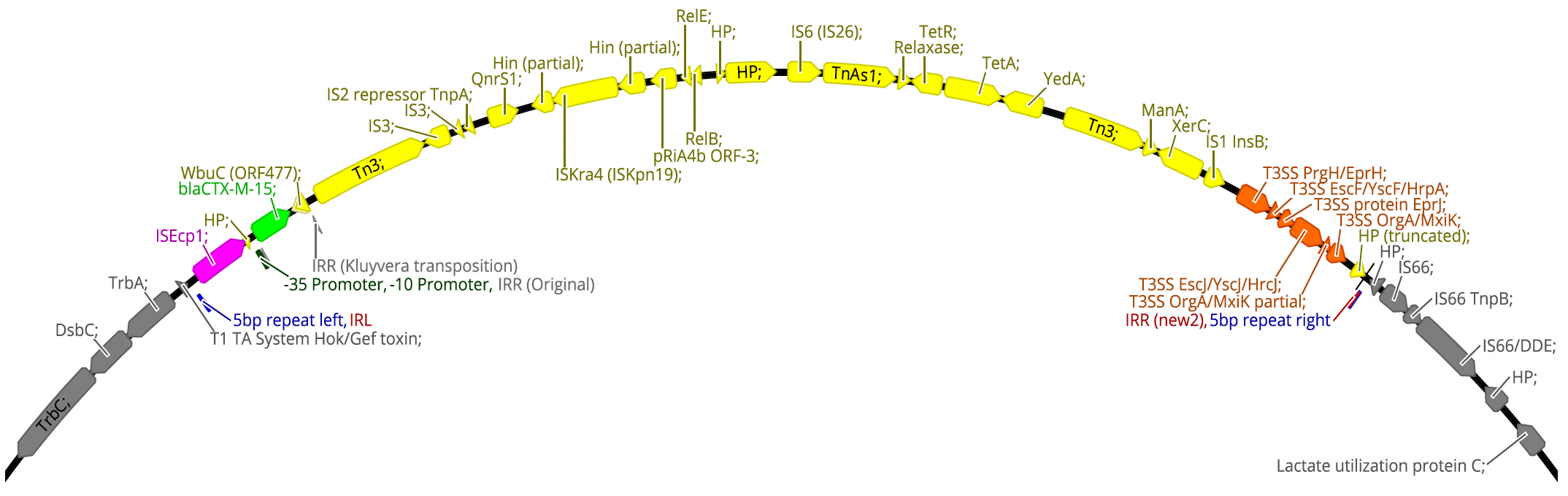 | **c** |
| **Supplementary** **Figure 1: Partial plasmid sequences of TTs 687CLOX128 with an IS*Ecp1* element of 27,093 bp in IncFIC (A), 961CLOX64 with an IS*Ecp1* element of 27,093 bp IncI1 (B) and 687AMP0.32 with an IS*Ecp1* element of 27,094 bp in IncFIC (C). The IS*Ecp1* elements each used the IR_L_ in combination with the IR_R(new2)_ which are shown in red, with the 5 bp repeats shown in blue denoting either end of the IS*Ecp1* element. In 687CLOX128 the interrupted gene *traC* at the insertion point at either end of the IS*Ecp1* element is shown in purple. The interrupted gene encoding a hypothetical protein (HP) at the insertion point at either end of the IS*Ecp1* element in 687AMP0.32 is also shown in purple. The IS*Ecp1* are all shown in pink, *bla*_CTX-M-15_ is shown in light green, the extra genes from the T3SS are shown in orange, the -35 and -10 promoters in dark green and any other genes within the IS*Ecp1* element are yellow. Any annotation in grey denotes plasmid DNA outside the boundaries of the IS*Ecp1* element.** | |

- 1. **Group 2: *ISEcp1* elements 10-15 kb in length inserted into conjugative plasmids**

Group 2 contained the four TTs: 687-N, 956AMP8, 956AMP16 and 961CLOX25.6 with IS*Ecp1* elements of 10-15 kb in length (**Supplementary** **Figure 2)** showing where the IS*Ecp1* elements had inserted into each of the plasmid backbones. There was gene loss from all these IS*Ecp1* elements compared to the donor strain IS*Ecp1*, again due to the recognition of a new imperfect IR_R_. Both 687-N and 961CLOX25.6 had lost the *tetAR* genes. **Supplementary** **Table 8** shows the imperfect IR_R_s. The TT 961CLOX25.6, was the only TT where IS*Ecp1* had inserted into the IncX4 plasmid during transposition from the parent 961. In the remaining TTs the IS*Ecp1* had inserted into a conjugative IncFIC plasmid. We cannot exclude insertion into other endogenous plasmids, but the predominant transconjugants were IncF1C plasmids, which may reflect a higher transfer rate of this plasmid type compared to other plasmids in the original donor strain. In 687-N, insertion of the IS*Ecp1* element had interrupted an alpha/beta hydrolase that was adjacent to *traX* and *finO* and overlapped at the end of pRiA4b ORF-3. In 961CLOX25.6 insertion of the IS*Ecp1* element had interrupted *virB8*, which forms part of the inner membrane complex of the type 4 secretion system (T4SS).

| **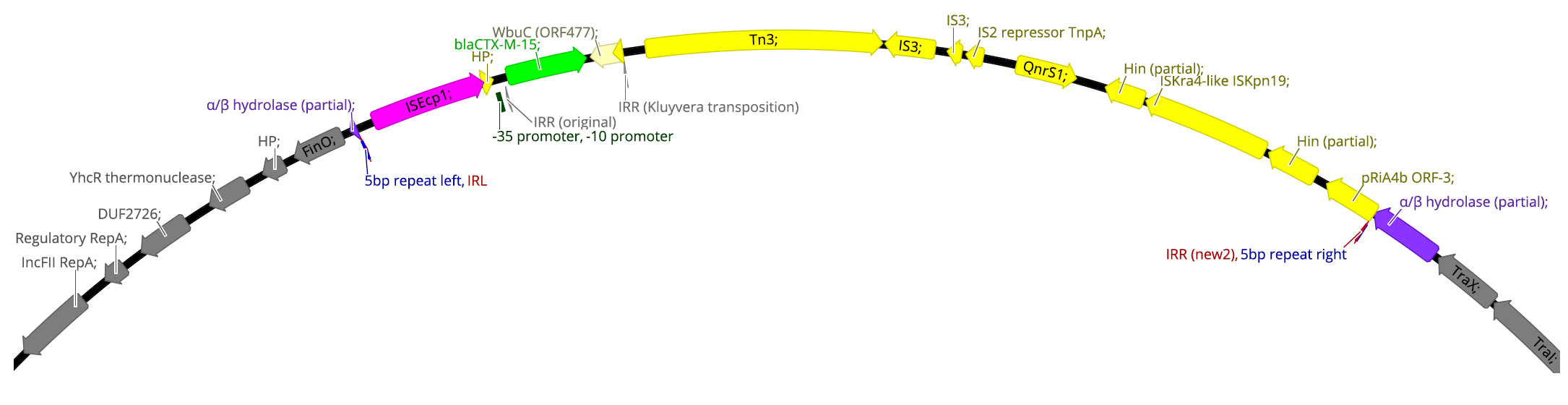** | **a** | **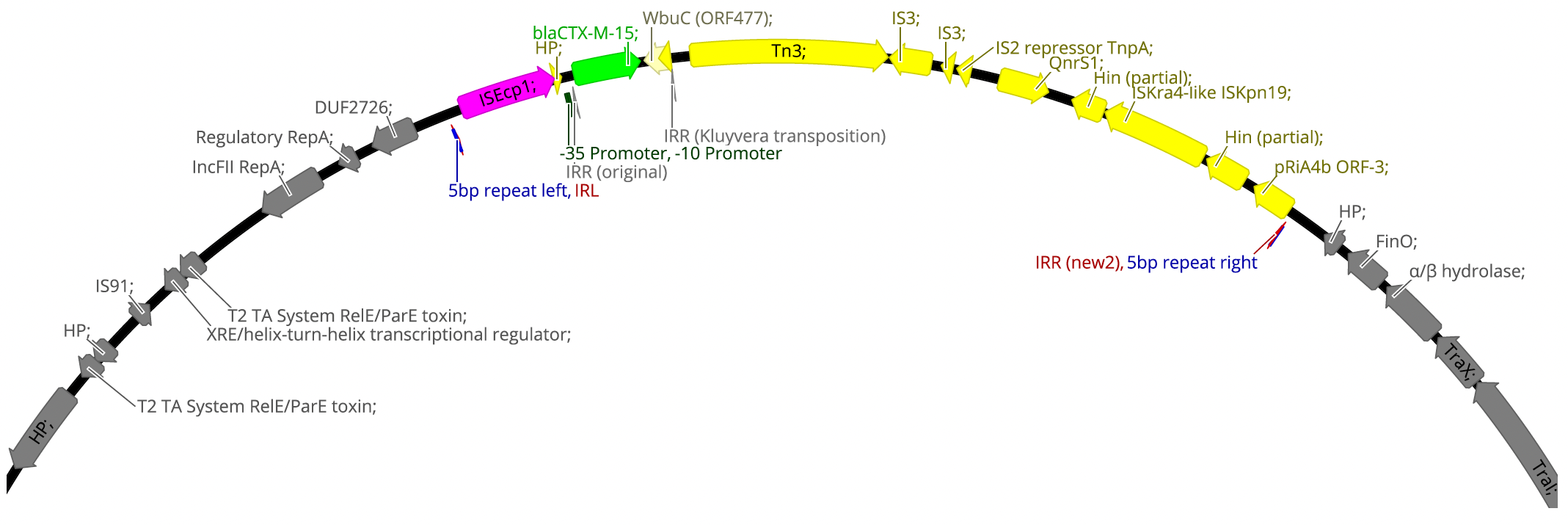** | **b** |
| --- | --- | --- | --- |
|  |  |  |  |
| **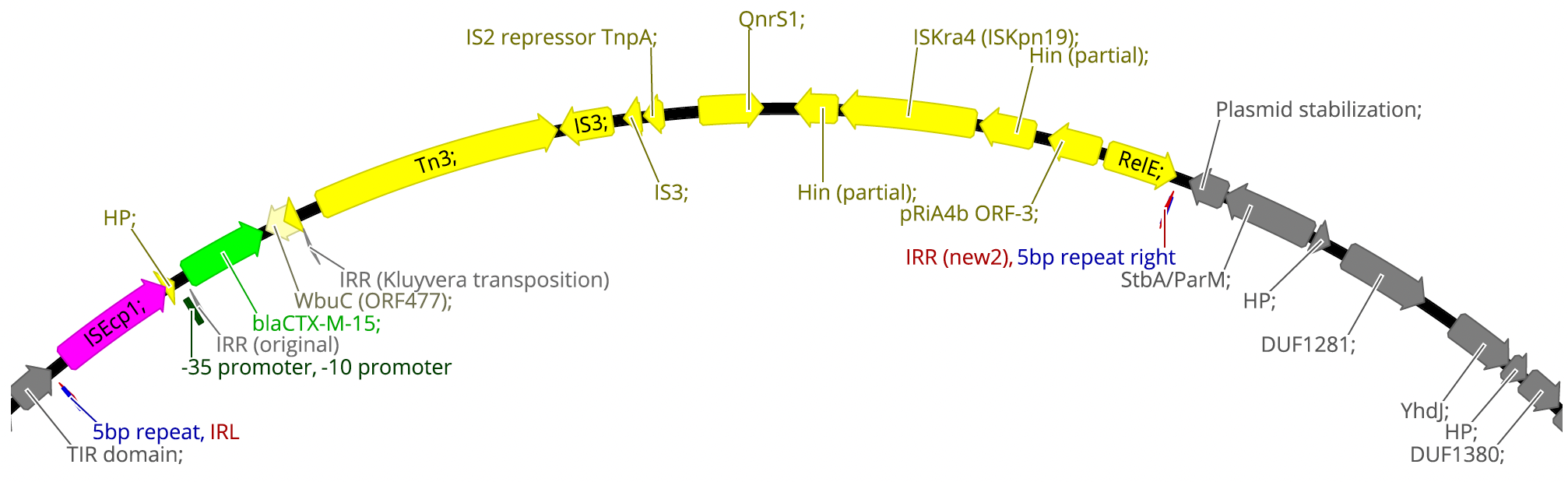** | **c** | **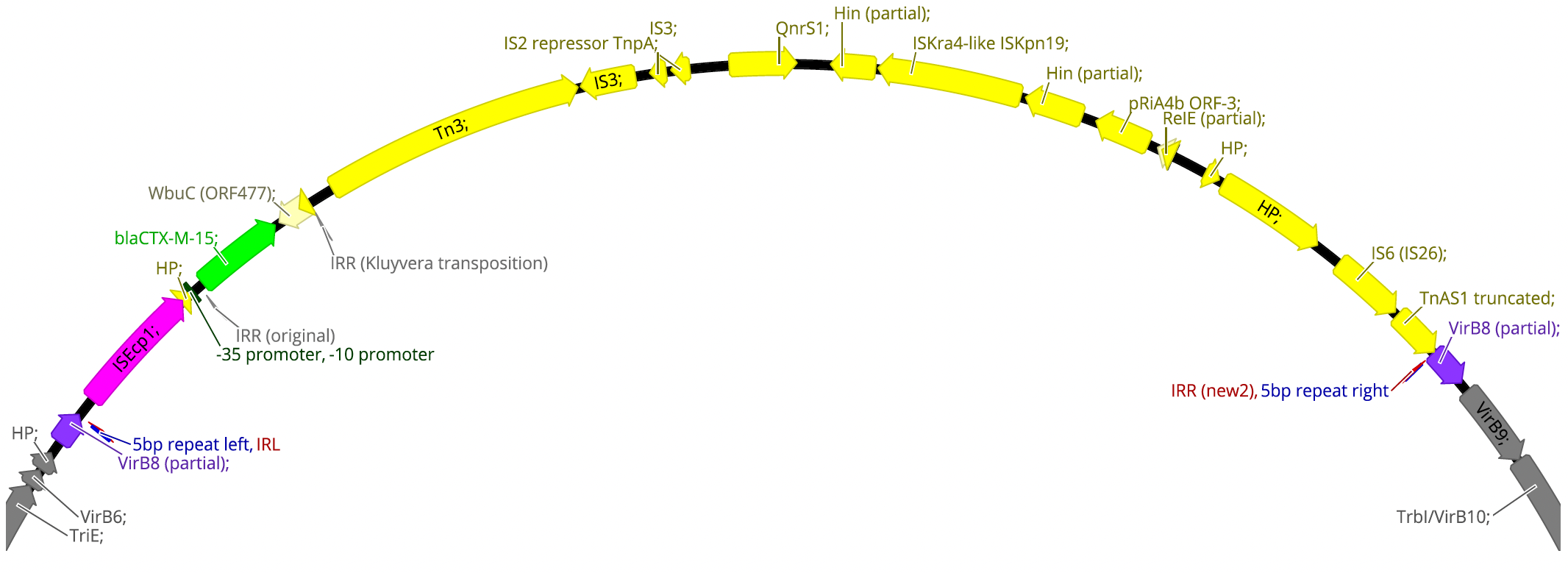** | **d** |
| **Supplementary** **Figure 2: The partial plasmid sequences of TTs 687-N with the insertion of the 11,394 bp IS*Ecp1* element in IncFIC (A), 956AMP8 with the insertion of the 11,394 bp IS*Ecp1* element in IncFIC (B), 956AMP16 with the insertion of the 12,196 bp IS*Ecp1* element in IncFIC (C) and 961CLOX25.6 with the insertion of the 14,722 bp IS*Ecp1* element in IncX4 (D). The IS*Ecp1* elements each used the IR_L_ in combination with the IR_R(new2)_ which are shown in red, with the 5 bp repeats shown in blue denoting either end of the IS*Ecp1* element. The interrupted gene in 687-N encoding an alpha/beta hydrolase at the insertion point at either end of the IS*Ecp1* element is shown in purple and the interrupted gene encoding *virB8* at the insertion point at either end of the IS*Ecp1* element in 961CLOX25.6 is also shown in purple. The IS*Ecp1* is shown in pink, *bla*_CTX-M-15_ is shown in light green, the -35 and -10 promoters in dark green and any other genes within the IS*Ecp1* element are yellow. Any annotation in grey denotes plasmid DNA outside the boundaries of the IS*Ecp1* element.** | | | |

- 1. **Group 3: IS*Ecp1* elements <10 kb in length inserted into conjugative plasmids**

Group 3 contained seven TTs (687AMP8, 876AMP8, 956-N, 876CAZ0.25, 956CLOX25.6, 956CLOX128, 687CLOX64) with IS*Ecp1* elements <10 kb in length. **Supplementary** **Figure 3** shows for each TT a section of the plasmid backbone where the IS*Ecp1* elements had inserted. The IS*Ecp1* element had inserted into the IncFIC (Sgro et al. 2019) plasmid in all but 876CAZ0.25, where it had inserted into the IncFII plasmid. These TTs showed almost total gene loss from the IS*Ecp1 bla*_CTX-M-15_ core genetic element, again likely due to imperfect IR_R_ recognition. Most of the IS*Ecp1* elements had no genes further downstream of *wbuC* and in 687AMP8, 876AMP8 and 956-N termination of the element occurred at IR_R(new2)_ resulting in truncation of *wbuC*. Only 687CLOX64 retained genes downstream of *wbuC*, which included Tn*3*, two IS*3*s, an IS*2* repressor TnpA and *qnrS1*. However, 687CLOX64 lost *tetAR* and all the remaining TTs had lost both *qnrS1* and *tetAR*.

Noted gene interruptions in the plasmids from IS*Ecp1* transposition were *traX* in 876AMP8, an alpha/beta hydrolase in 956CLOX25.6 (as seen in 687-N) and *traD* in 876CAZ0.25 resulting in the stop codon being separated from the coding sequence.

| **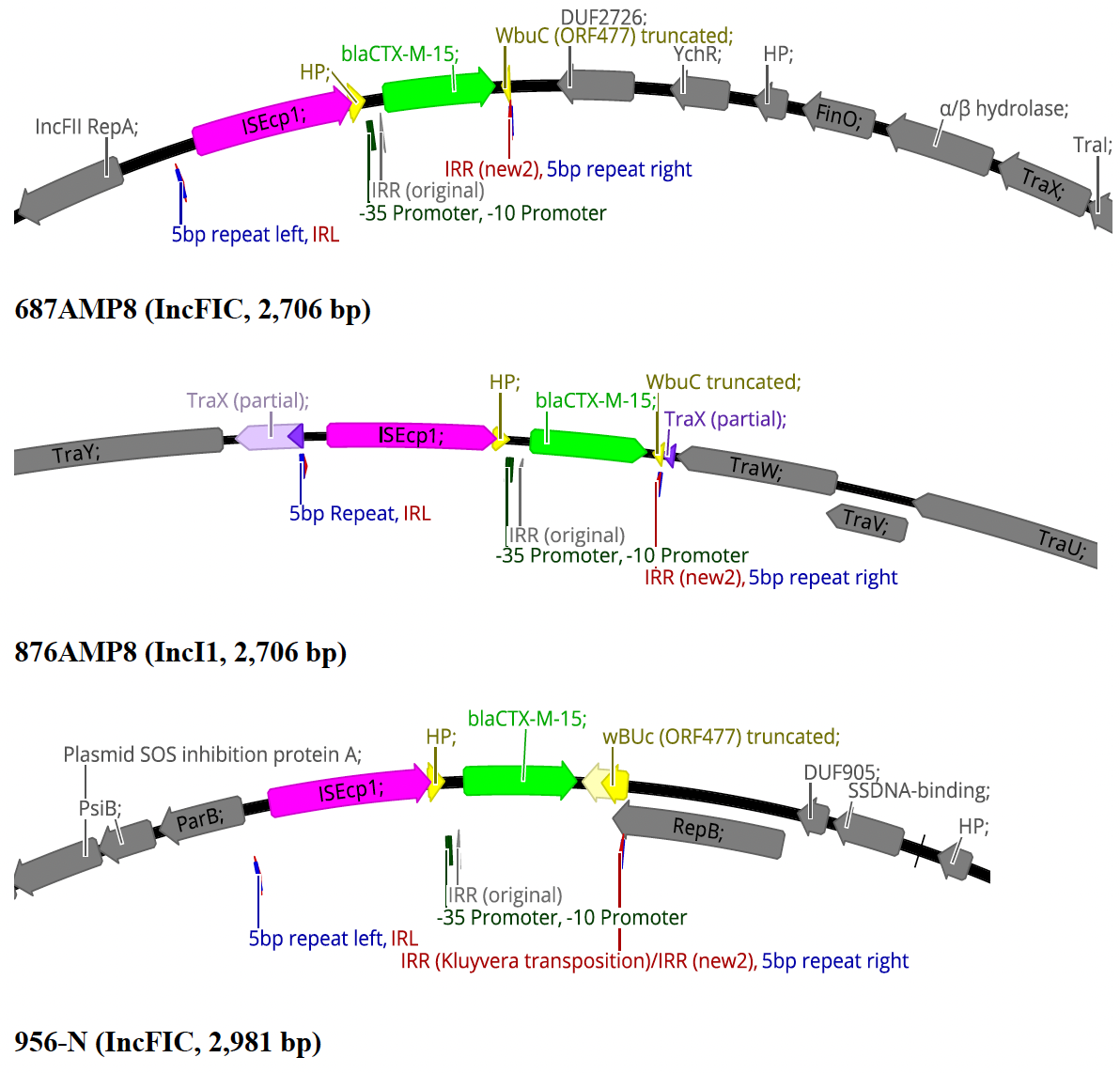 a**  **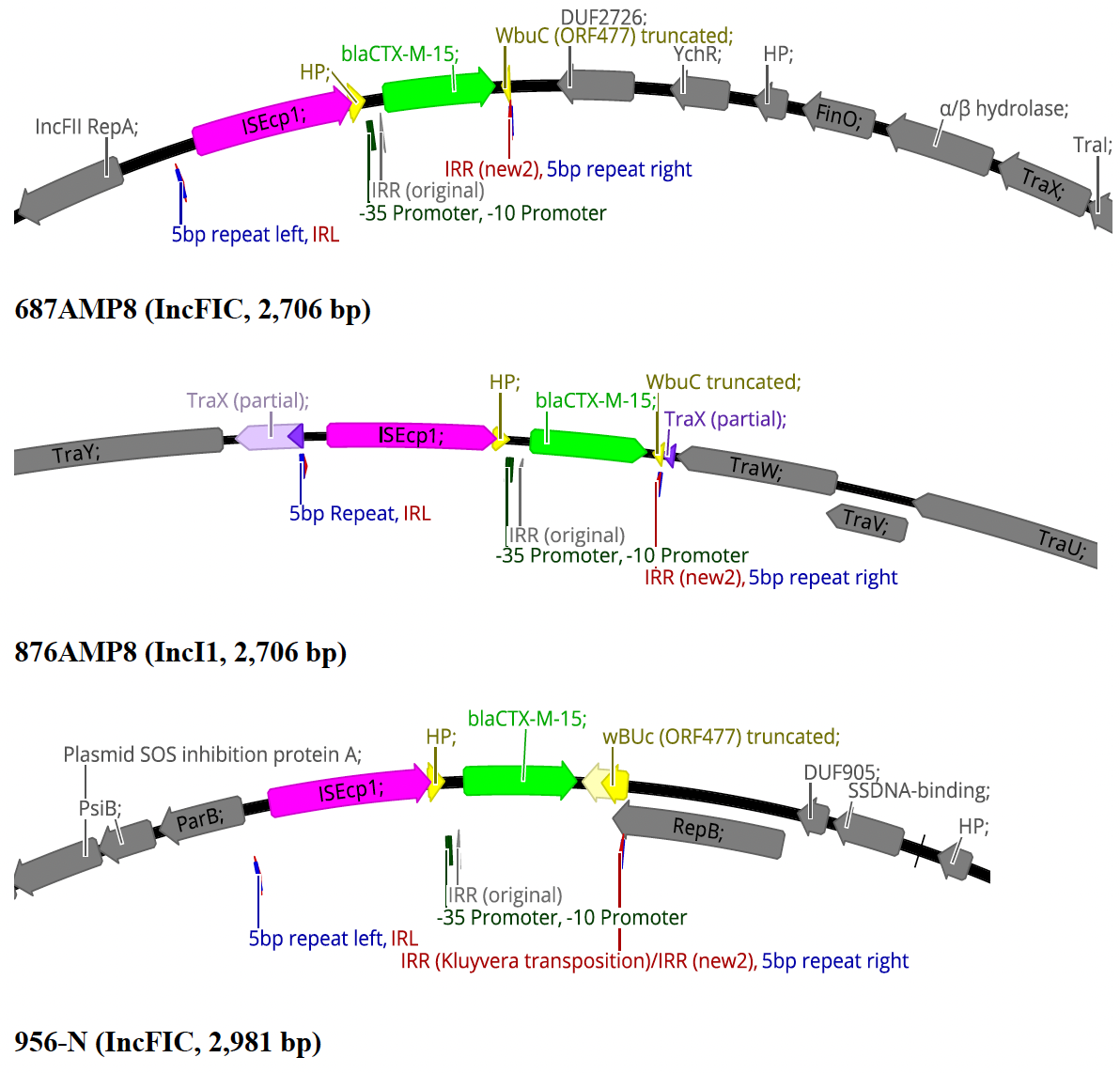b**  **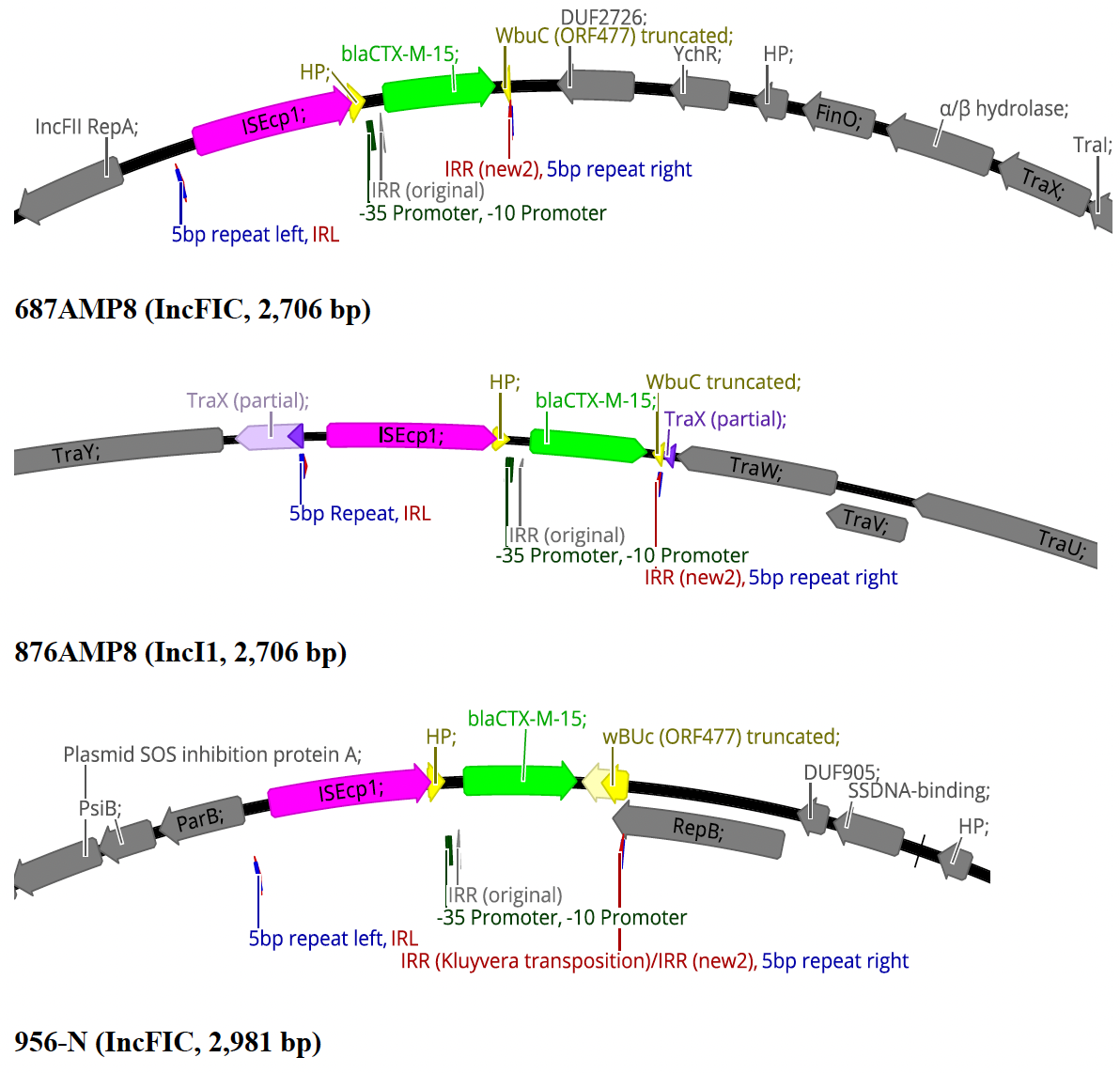c** | **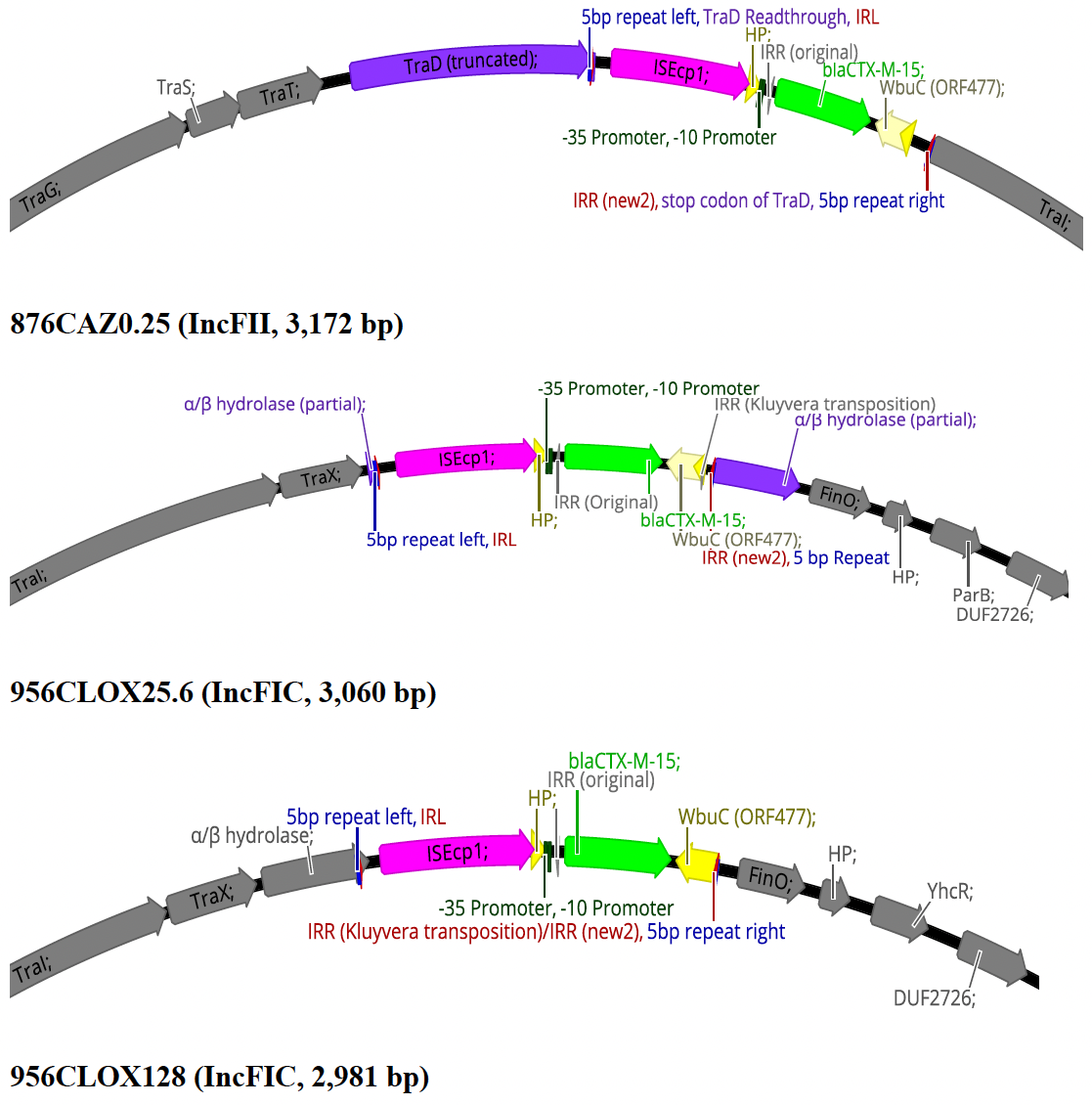 d**  **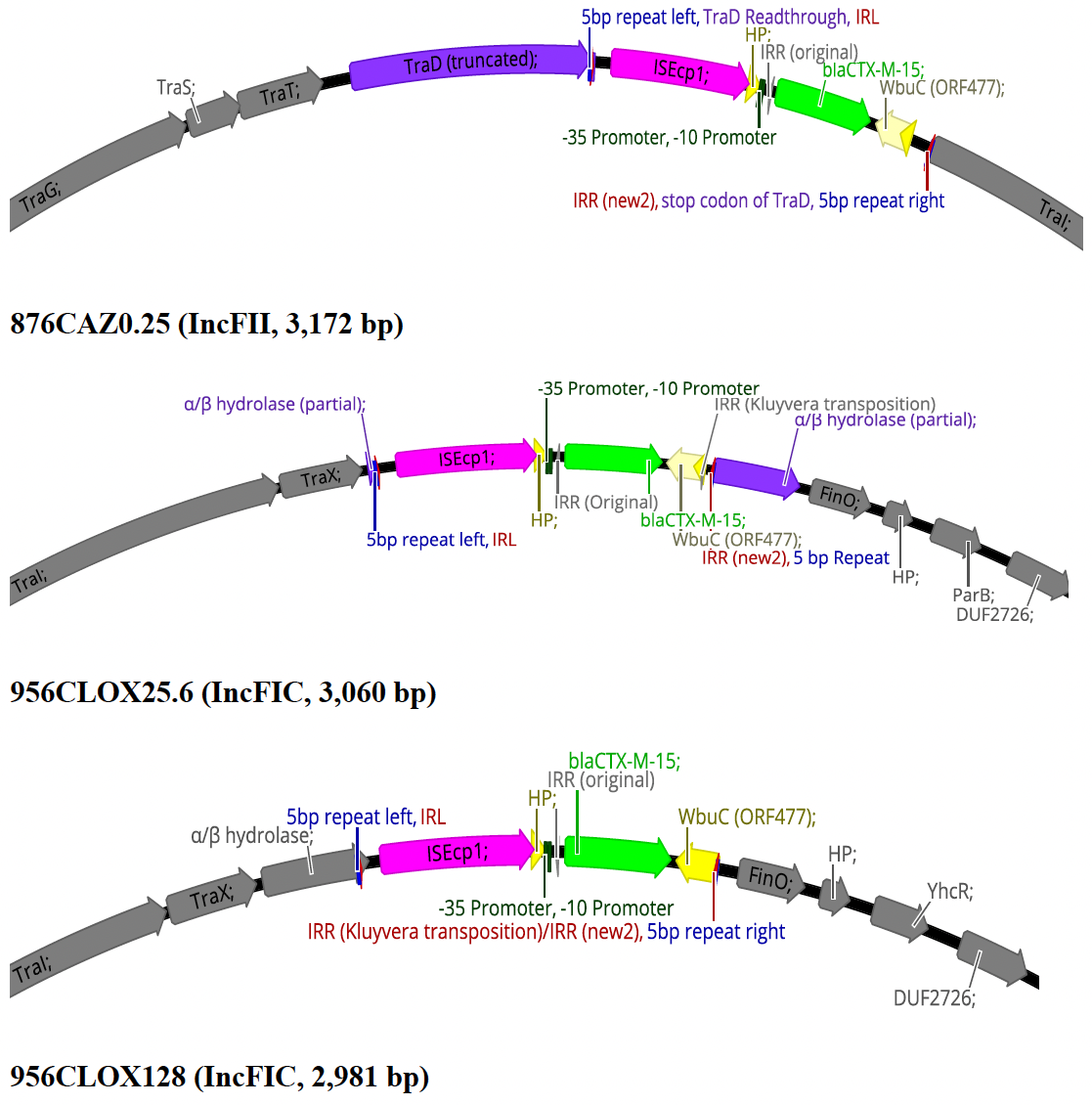 e**  **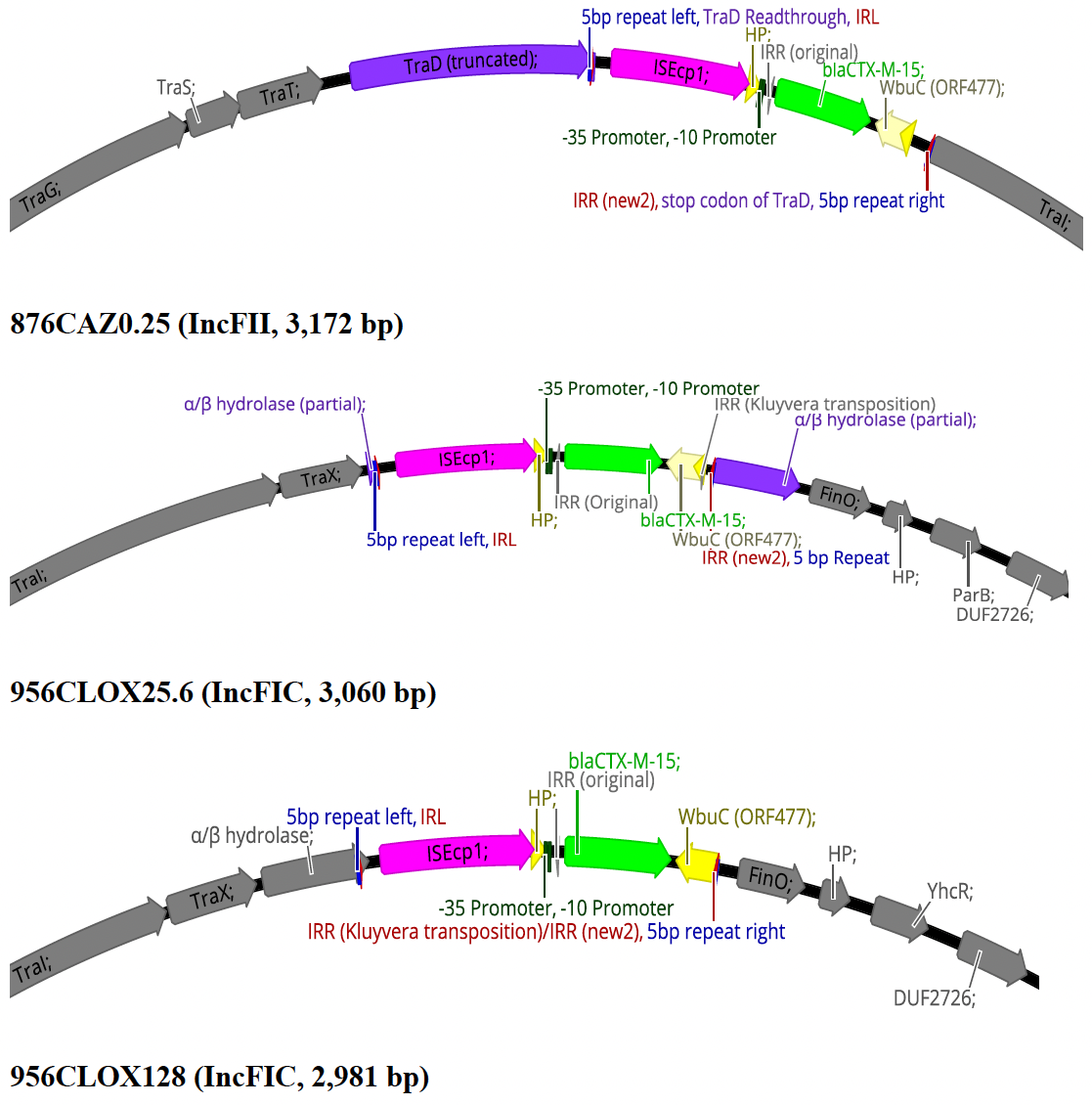 f**  **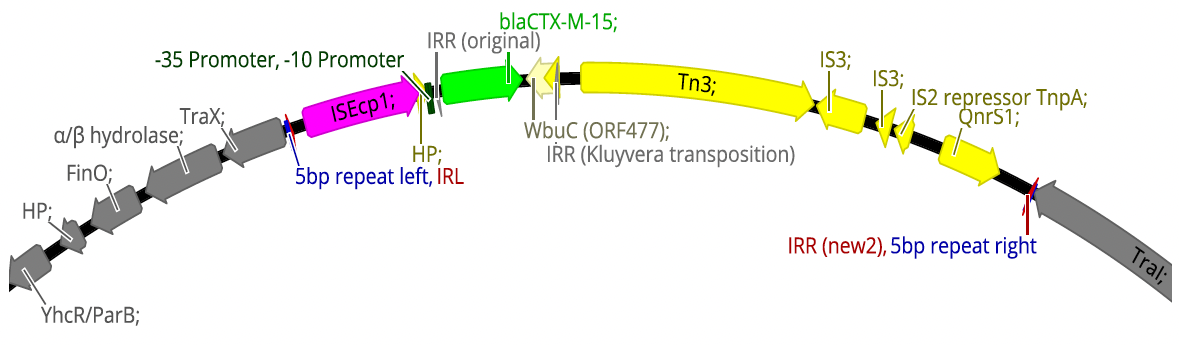 g** |
| --- | --- |
| **Supplementary** **Figure 3: The partial plasmid sequences of TTs 687AMP8 with the insertion of the 2,706 bp IS*Ecp1* element in IncFIC (A), 876AMP8 with the insertion of the 2,706 bp IS*Ecp1* element in IncI1 (B), 956-N with the insertion of the 2,981 bp IS*Ecp1* element in IncFIC (C), which all had a truncated *wbuC*. The partial plasmid backbones of TTs 876CAZ0.25 with the insertion of the 3,172 bp IS*Ecp1* element in IncFII (D), 956CLOX25.6 with the insertion of the 3,060 bp IS*Ecp1* element in IncFIC (E) and 956CLOX128 with the insertion of the 2,981 bp IS*Ecp1* element in IncFIC (F) which all had similar IS*Ecp1* genetic environments with no further genes downstream of *wbuC*. The plasmid backbone of TT 687CLOX64 showing the insertion of the 8,284 bp IS*Ecp1* element in IncFIC (G) which had only 5 genes downstream from *wbuC*. The IR_L_ used in combination with the IR_R(new2)_ on each figure are both shown in red, with the 5 bp repeats shown in blue which denote either end of the IS*Ecp1* element. Any interrupted genes are shown in purple. In all plasmid backbones, the IS*Ecp1* is shown in pink, *bla*_CTX-M-15_ is shown in light green, the -35 and -10 promoters are shown in dark green and any other genes within the IS*Ecp1* element are yellow. Any annotation in grey denotes plasmid DNA outside the boundaries of the IS*Ecp1* element.** | |

- 1. **IS*Ecp1* insertion sites were similar in the IncFIC plasmid backbone of different strains.**

There were some similarities in where IS*Ecp1* had inserted into the plasmid backbone. Ten out of 15 of the IS*Ecp1* elements were located on the IncFIC plasmid. The IncFIC plasmids of the parents were identical at 61,878 bp except strain 956 where it was 11 bp smaller at 61,867 bp. This was due to a CTGCTA repeating region within a HP protein, that was repeated 20 times in all of the IncFIC plasmids except 956 where it was only repeated 18 times. This may have been a sequencing artifact or an assembly error due to the repeating region. All but two of the IS*Ecp1* variants had inserted into the same general region of the plasmid, which included the genes *traI*, *traX*, an alpha/beta hydrolase, *finO*, a hypothetical protein (HP), *yhcR*, DUF2726, *repA*, IncFIC RepA, *relE*/*parE*, xenobiotic response element (XRE) transcriptional regulator, IS91, a HP, *relE*/*parE*, a HP, toll-interleukin-1 receptor (TIR) domain and a gene encoding a plasmid stabilisation protein. This was similar to that seen for the IncFII plasmid, (TT 876CAZ0.25) where it had interrupted *traD* immediately adjacent to *traI*. In only two cases (TTs 961CLOX64 and 876AMP8) did the IS*Ecp1* elements insert into different locations in the resident conjugative IncI1 plasmid, so it is unclear whether there was a pattern of insertion. Only in TT 961CLOX25.6 was the IS*Ecp1* element inserted into the resident conjugative IncX4 plasmid).

To compare the insertion sites in greater detail for the IncFIC plasmids, a plasmid map was constructed using Geneious Prime from the WGS of the parent strain 687. **Supplementary** **Figure 4** shows a plasmid map of the IncFIC plasmid and identifies on it the three regions utilised as insertion sites, with the green region encoding *repB* where the IS*Ecp1* in TT 956-N had inserted, the pink region encoding *traC* where the IS*Ecp1* in TT 687CLOX128 had inserted and blue indicating the large genomic region where the remaining *ISEcp1* elements had inserted in 687AMP0.32, 687-N, 956AMP8, 956AMP16, 687AMP8, 956-N, 956CLOX25.6, 956CLOX128 and 687CLOX64. For 876CAZ0.25 the *traD* gene is highlighted in orange in **Supplementary** **Figure 4**, to show where IS*Ecp1* had inserted into the IncFII plasmid, which had a very similar plasmid backbone to the IncFIC plasmid, with the region of interest for this analysis identical.


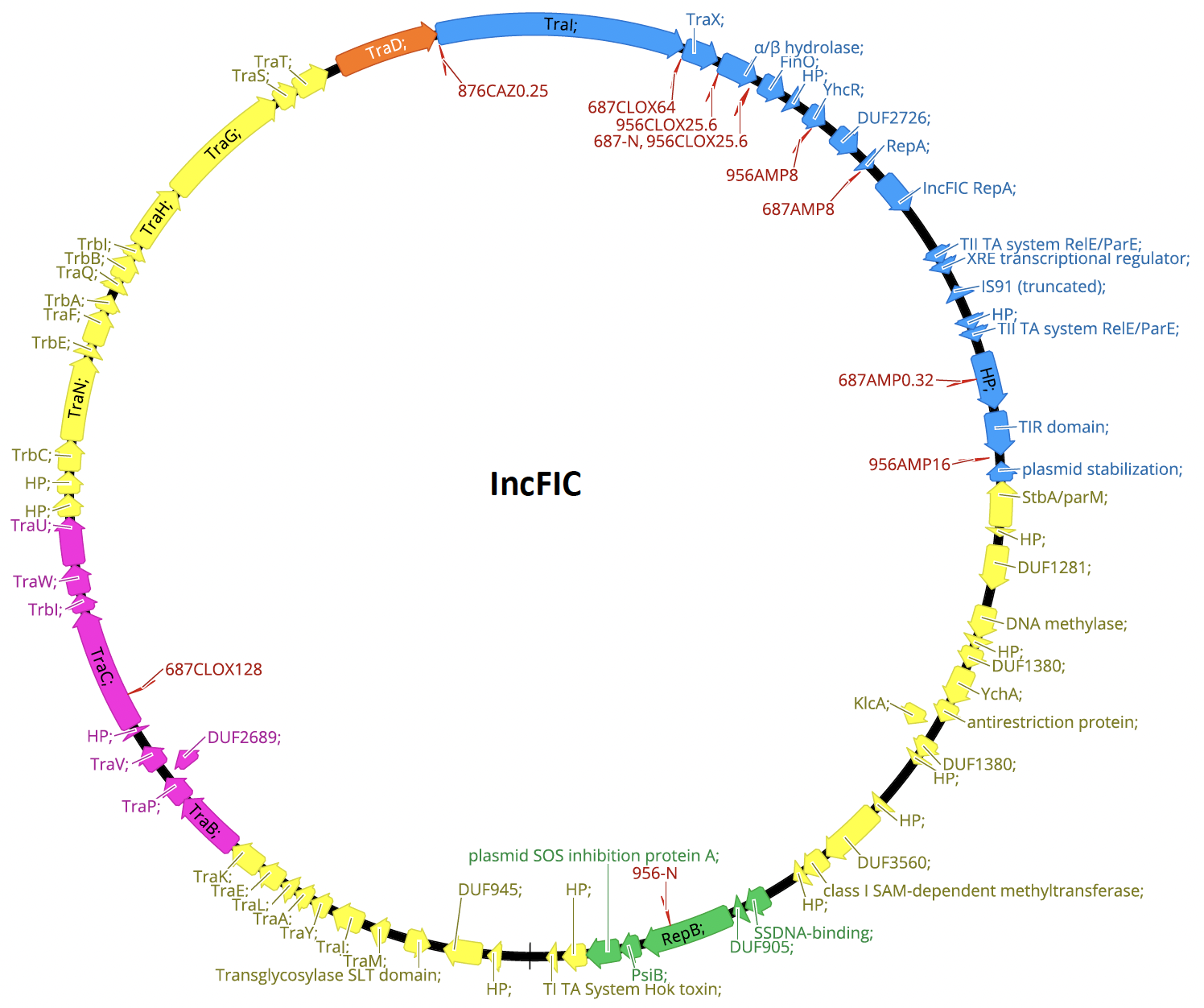


**Supplementary** **Figure 4: The plasmid map of the IncFIC plasmid from the parent strains, which was 61, 878 bp in 687 and 961 and 61, 867 bp in 956. The plasmid map details the insertion locations seen within the TTs as red annotations of the TT names and the main regions of IS*Ecp1* insertion. The main regions of insertion of IS*Ecp1* identified were the green region indicating TT 956-N, the pink region indicating TT 687CLOX128 and the blue region indicating the insertion region used by the remaining IS*Ecp1* elements found in an IncFIC plasmid. In addition, the *traD* gene is highlighted in orange on this plasmid map to show where 876CAZ0.25 had inserted into the similarly structured IncFII plasmid.**

| **Table S1: The 39 *E. coli* EVAL farms dairy farm isolates sampled between 2017-18.** | | | |  |
| --- | --- | --- | --- | --- |
| **Isolate Name** | **Isolation Date** | **Sampling Location** | **Selective Media** | **Disc Diffusion Assay Resistance Profile** |
| EcoSL1010-687 | 10/10/2017 | Slurry Tank | CA*🡪TBX* | AMP, CAZ, CTX, CPD, ATM, (STREP), TET, (CIP), AZM |
| EcoSL1710-726 | 17/10/2017 | Slurry Tank | Mac*+CTX*🡪TBX+CTX | AMP, CAZ, CTX, CPD, ATM, (STREP), TET, CIP, (NAL) |
| EcoSL3110-774 | 31/10/2017 | Slurry Tank | Mac+CTX🡪TBX+CTX | AMP, CAZ, CTX, CPD, ATM, (STREP), TET, (CIP) |
| EcoHS11212-873 | 12/12/2017 | Heifer shed 1 (older cows) | CA🡪TBX+CTX | AMP, CAZ, CTX, CPD, ATM, TET, (CIP), (NAL) |
| EcoHS11212-874 | 12/12/2017 | Heifer shed 1 (older cows) | CA🡪TBX+CTX | AMP, CAZ, CTX, CPD, ATM, (STREP), TET, (CIP) |
| EcoHS11212-875 | 12/12/2017 | Heifer shed 1 (older cows) | CA🡪TBX+CTX | AMP, CAZ, CTX, CPD, ATM, (STREP), TET, (CIP), (NAL), AZM |
| EcoHS11212-876 | 12/12/2017 | Heifer shed 1 (older cows) | CA🡪TBX+CTX | AMP, FOX, CAZ, CTX, CPD, (CIP), AZM |
| EcoHS11212-877 | 12/12/2017 | Heifer shed 1 (older cows) | CA🡪TBX+CTX | AMP, CAZ, CTX, CPD, ATM, (STREP), TET, (CIP), (NAL) |
| EcoHS11212-878 | 12/12/2017 | Heifer shed 1 (older cows) | CA🡪TBX+CTX | AMP, CAZ, CTX, CPD, ATM, (STREP), TET, (CIP), AZM |
| EcoHS11212-879 | 12/12/2017 | Heifer shed 1 (older cows) | CA🡪TBX+CTX | AMP, CAZ, CTX, CPD, ATM, TET, (CIP) |
| EcoHS11212-880 | 12/12/2017 | Heifer shed 1 (older cows) | CA🡪TBX+CTX | AMP, CAZ, CTX, CPD, ATM, (STREP), TET, (CIP), AZM |
| EcoHS11212-881 | 12/12/2017 | Heifer shed 1 (older cows) | CA🡪TBX+CTX | AMP, CAZ, CTX, CPD, ATM, TET, (CIP), AZM |
| EcoMHE1212-939 | 12/12/2017 | Muck heap effluent | CA🡪TBX+CTX | AMP, CAZ, CTX, CPD, ATM, (STREP), TET, (CIP) |
| EcoMHE1212-940 | 12/12/2017 | Muck heap effluent | CA🡪TBX+CTX | AMP, CAZ, CTX, CPD, ATM, (STREP), TET, (CIP), (NAL) |
| EcoMHE1212-941 | 12/12/2017 | Muck heap effluent | CA🡪TBX+CTX | AMP, CAZ, CTX, CPD, ATM, (STREP), TET, (CIP), (NAL) |
| EcoMHE1212-942 | 12/12/2017 | Muck heap effluent | CA🡪TBX+CTX | AMP, CAZ, CTX, CPD, ATM, (STREP), TET, (CIP), (NAL) |
| EcoMHE1212-944 | 12/12/2017 | Muck heap effluent | CA🡪TBX+CTX | AMP, CAZ, CTX, CPD, ATM, STREP, TET, (CIP) |
| EcoMHE1212-945 | 12/12/2017 | Muck heap effluent | CA🡪TBX+CTX | AMP, CAZ, CTX, CPD, ATM, (STREP), TET, (CIP), (NAL) |
| EcoMHE1212-946 | 12/12/2017 | Muck heap effluent | CA🡪TBX+CTX | AMP, CAZ, CTX, CPD, ATM, (STREP), TET, (CIP), (NAL) |
| EcoMHE1212-947 | 12/12/2017 | Muck heap effluent | CA🡪TBX+CTX | AMP, CAZ, CTX, CPD, ATM, (STREP), TET, (CIP), (NAL) |
| EcoMHE1212-948 | 12/12/2017 | Muck heap effluent | CA🡪TBX+CTX | AMP, CAZ, CTX, CPD, ATM, (STREP), TET, (CIP), (NAL), AZM |
| EcoMHE1212-949 | 12/12/2017 | Muck heap effluent | CA🡪TBX+CTX | AMP, CAZ, CTX, CPD, ATM, (STREP), TET, (CIP), (NAL), AZM |
| EcoMHE1801-950 | 18/01/2018 | Muck heap effluent | CA🡪TBX+CTX | AMP, CAZ, CTX, CPD, ATM, (STREP), (CIP), (NAL) |
| EcoMHE1801-951 | 18/01/2018 | Muck heap effluent | CA🡪TBX+CTX | AMP, CAZ, CTX, CPD, ATM, TET, (CIP), (NAL) |
| EcoMHE1801-952 | 18/01/2018 | Muck heap effluent | CA🡪TBX+CTX | AMP, CAZ, CTX, CPD, ATM, TET, (CIP) |
| EcoMHE1801-953 | 18/01/2018 | Muck heap effluent | CA🡪TBX+CTX | AMP, CAZ, CTX, CPD, ATM, TET, (CIP) |
| EcoMHE1801-955 | 18/01/2018 | Muck heap effluent | CA🡪TBX+CTX | AMP, CAZ, CTX, CPD, ATM, TET, (CIP) |
| EcoMHE1801-956 | 18/01/2018 | Muck heap effluent | CA🡪TBX+CTX | AMP, CAZ, CTX, CPD, ATM, (STREP), (CIP), (NAL), (CHLOR), AZM |
| EcoMHE1801-957 | 18/01/2018 | Muck heap effluent | CA🡪TBX+CTX | AMP, CAZ, CTX, CPD, ATM, (STREP), TET, (CIP) |
| EcoSS2501-958 | 25/01/2018 | Slurry solids | CA🡪TBX+CTX | AMP, CAZ, CTX, CPD, ATM, (STREP), TET, (CIP) |
| EcoSS2501-959 | 25/01/2018 | Slurry solids | CA🡪TBX+CTX | AMP, CAZ, CTX, CPD, ATM, (STREP), TET, (CIP) |
| EcoSS2501-960 | 25/01/2018 | Slurry solids | CA🡪TBX+CTX | AMP, CAZ, CTX, CPD, ATM, (STREP), TET, (CIP) |
| EcoSS2501-961 | 25/01/2018 | Slurry solids | CA🡪TBX+CTX | AMP, CAZ, CTX, CPD, ATM, (IMP), (STREP), TET, (CIP), AZM |
| EcoSS2501-962 | 25/01/2018 | Slurry solids | CA🡪TBX+CTX | AMP, CAZ, CTX, CPD, ATM, (STREP), TET, (CIP), SXT, AZM |
| EcoSS2501-963 | 25/01/2018 | Slurry solids | CA🡪TBX+CTX | AMP, CAZ, CTX, CPD, ATM, STREP, TET, (CIP) |
| EcoSS2501-964 | 25/01/2018 | Slurry solids | CA🡪TBX+CTX | AMP, CAZ, CTX, CPD, ATM, TET |
| EcoSS2501-965 | 25/01/2018 | Slurry solids | CA🡪TBX+CTX | AMP, CAZ, CTX, CPD, ATM, TET, (CIP) |
| EcoSS2501-966 | 25/01/2018 | Slurry solids | CA🡪TBX+CTX | AMP, CAZ, CTX, CPD, ATM, (STREP), TET, (CIP), (NAL), AZM |
| EcoSS2501-967 | 25/01/2018 | Slurry solids | CA🡪TBX+CTX | AMP, CAZ, CTX, CPD, ATM, STREP, TET, (CIP), (NAL) |

***Footnote for Table S1****: Mac – MacConkey Agar, CTX – cefotaxime (at a working concentration of 2 mg L^-1^), TBX - Tryptone Bile X-Glucuronide Agar, CA – CHROMagar ESBL. The 🡪 signifies the isolate was initially isolated on one medium and was then restreaked onto a second medium for E. coli confirmation. Antibiotics in brackets denote an intermediate result from the disc diffusion assay. Antibiotic abbreviations: AMP (Ampicillin), FOX (Cefoxitin), CAZ (Ceftazidiime), CTX (Cefotaxime), CPD (Cefpodoxime), ATM (Aztreonam), IMP (Imipenem), STREP (Streptomycin), TET (Tetracycline), CIP (Ciprofloxacin), NAL (Nalidixic Acid), SXT (Trimethoprim/sulfamethoxazole), CHLOR (Chloramphenicol) and AZM (Azithromycin).*

| **Table S2: Antibiotic stocks for minimum inhibitory concentration agar dilution. Detailed are the potency (as defined by the supplier), stock concentration, solvent and concentration range tested. All stocks were prepared as a g L^-1^ concentration unless stated otherwise.** | | | | | | | | |
| --- | --- | --- | --- | --- | --- | --- | --- | --- |
| **Antibiotic** | **Abbrv** | **Supplier** | **Potency (µg/mg)** | **Stock Concentrations** | | | **Solvent** | **Concentration Range (mg L^-1^)** |
|  |  |  |  | **Stock 1** | **Stock 2** | **Stock 3** |  |  |
| Ampicillin | AMP | Sigma | 916 | 40 | 4 | 0.4 | Water | 4-512 |
| Co-Amoxiclav (Amoxicillin and Clavulanic Acid) 4:1 | AMC | Sigma | 992 | 1 | - | - |  | 4-512 |
| Cefoxitin | FOX | Cayman Chemical Company | 980 | 10 | 1 | 0.1 | Water | 0.032-512 |
| Cefotaxime | CTX | Sigma | 964 | 2 | 0.2 | 0.02 | Water | 0.25-512 |
| Ceftazidime | CAZ | MedChem Express | 990 | 2 | 0.2 | 0.02 | Water | 0.5-512 |
| Cefpodoxime | CPD | Cayman Chemical Company | 950 | 2 | 0.2 | 0.02 | Ethanol | 0.25-512 |
| Cefquinome | CFQ | Chem Cruz | 900 | 2 | 0.2 | 0.02 | Water | 0.125-512 |
| Aztreonam | ATM | Cayman Chemical Company | 950 | 2 | 0.2 | 0.02 | Ethanol | 0.25-512 |
| Imipenem | IMP | Sigma | 885 | 2 | 0.2 | 0.02 | Water | 0.5-8 |
| Ertapenem | ERT | Sigma | 843 | 2 | 0.1 | 0.01 | Water | 0.032-1 |
| Meropenem | MPM | Sigma | 702 | 2 | 0.1 | 0.01 | Water | 0.064-2 |
| Streptomycin | STREP | Sigma | 732 | 40 | 4 | 0.4 | Water | 4-64 |
| Gentamicin | GEN | Sigma | 590 | 2 | 0.2 | - | Water | 1-8 |
| Neomycin | NEO | Alfa Aesar | 600 | 40 | 4 | 0.4 | Water | 4-32 |
| Apramycin | APR | Sigma | 450 | 40 | 4 | 0.4 | Water | 4-128 |
| Tobramycin | TOB | Sigma | 900 | 2 | 0.2 | - | Water | 1-8 |
| Tetracycline | TET | Sigma | 980 | 40 | 4 | 0.4 | Ethanol | 2-512 |
| Tigecycline | TIG | Sigma | 992 | 2 | 0.2 | 0.02 | DMSO | 0.25-2 |
| Ciprofloxacin | CIP | Sigma | 980 | 2 | 0.1 | 0.01 | 0.1M Acetic Acid | 0.125-2 |
| Enrofloxacin | ENR | Sigma | 990 | 2 | 0.1 | 0.01 | DMSO | 0.032-64 |
| Nalidixic Acid | NAL | Sigma | 900 | 6 | - | - | Water | 0.032-512 |
| SXT | SXT | Sigma | 980 | 2 | 0.2 | 0.02 | DMSO | 0.5-16 |
| Chloramphenicol | CHLOR | Sigma | 980 | 2 | 0.2 | 0.02 | Ethanol | 4-32 |
| Azithromycin | AZM | Chem Cruz | 958 | 40 | 4 | 0.4 | DMSO | 8-64 |
| Colistin | COL | Sigma | 753 | 2 | 0.2 | 0.02 | Water | 1-8 |
| Nitrofurantoin | NIT | Sigma | 980 | 10 | - | - | Ethanol | 32-256 |
| ***Footnote for Table S2****: *Cloxacillin was only utilised when conducting MICs on the transposition transconjugants (TT)* | | | | | | | | |

| **Table S3: Antibiotic disc concentrations, disc supplier and zone clearing breakpoints of the antibiotic discs used within disc diffusion assays utilised by Baker et al (2022).** | | | | | | | |
| --- | --- | --- | --- | --- | --- | --- | --- |
| **Antibiotic Class** | **Name** | **Abbrv.** | **Disc Conc (µg)** | **Zone Clearing Sizes (mm)** | | | **Disc Supplier** |
|  |  |  |  | **Res** | **Interm** | **Susc** |  |
| β-Lactam | Ampicillin | AMP | 10 | >17 | 14-16 | <13 | ProLab |
| β-Lactam/β-Lactamase Inhibitor | Amoxicillin/Clavulanic Acid | AMC | 20/10 | >18 | 14-17 | <13 | ProLab |
| 2^nd^ Generation Ceph | Cefoxitin | FOX | 30 | >18 | 15-17 | <14 | ProLab |
| 3^rd^ Generation Ceph | Ceftazidime | CAZ | 30 | >21 | 18-20 | <17 | ProLab |
| 3^rd^ Generation Ceph | Cefotaxime | CTX | 30 | >26 | 23-25 | <22 | ProLab |
| 3^rd^ Generation Ceph | Ceftiofur | EFT | 30 | >23 | 20-22 | <19 | Oxoid |
| 3^rd^ Generation Ceph | Cefpodoxime | CPD | 10 | >21 | 18-20 | <17 | ProLab |
| 4^th^ Generation Ceph | Cefquinome | CFQ | 30 | >23 | 20-22 | <19 | Bioconnections |
| Monobactam | Aztreonam | ATM | 30 | >21 | 18-20 | <17 | ProLab |
| Carbapenem | Imipenem | IMP | 10 | >23 | 20-22 | <19 | ProLab |
| Aminoglycoside | Streptomycin | STREP | 10 | >15 | 12-14 | <11 | ProLab |
| Tetracyclines | Tetracycline | TET | 30 | >15 | 12-14 | <11 | ProLab |
| Fluoroquinolones | Ciprofloxacin | CIP | 5 | >21 | 16-20 | <15 | ProLab |
| Fluoroquinolones | Enrofloxacin | ENR | 5 | >26 | 19-25 | <18 | Oxoid |
| Quinolone | Nalidixic Acid | NAL | 30 | >19 | 14-18 | <13 | ProLab |
| Folate Pathway Inhibitors | Sulphonamides | SULP | 300 | >17 | 13-16 | <12 | Oxoid |
| Folate Pathway Inhibitors | Trimethoprim-Sulfamethoxazole | SXT | 1.25/23.75 | >16 | 11-15 | <10 | ProLab |
| Phenicols | Chloramphenicol | CHLOR | 30 | >18 | 13-17 | <12 | ProLab |
| Nitrofurans | Nitrofurantoin | NIT | 300 | >17 | 13-16 | <12 | ProLab |
| Macrolides | Azithromycin | AZM | 15 | >13 | 12-14 | <11 | ProLab |

| **Supplementary S4: Bioproject PRJNA1196928 showing the breakdown of the Accession numbers and their corresponding, Isolate Codes, Assembly IDs and WGS IDs within the Bioproject number.** | | | |
| --- | --- | --- | --- |
| **Assembly ID** | **WGS ID** | **Accession Number** | **Isolate Code** |
| GCA_046246225.1 | JBJYNF000000000 | SAMN45706860 | EcoSL1710-726 |
| GCA_046246205.1 | JBJYNG000000000 | SAMN45706859 | EcoSL1010-687 |
| GCA_046246175.1 | JBJYNE000000000 | SAMN45706861 | EcoSL3110-774 |
| GCA_046246155.1 | JBJYND000000000 | SAMN45706862 | EcoHS11212-873 |
| GCA_046246115.1 | JBJYNA000000000 | SAMN45706865 | EcoHS11212-876 |
| GCA_046246125.1 | JBJYNC000000000 | SAMN45706863 | EcoHS11212-874 |
| GCA_046246105.1 | JBJYNB000000000 | SAMN45706864 | EcoHS11212-875 |
| GCA_046246055.1 | JBJYMZ000000000 | SAMN45706866 | EcoHS11212-877 |
| GCA_046246045.1 | JBJYMY000000000 | SAMN45706867 | EcoHS11212-878 |
| GCA_046246025.1 | JBJYMW000000000 | SAMN45706869 | EcoHS11212-880 |
| GCA_046246035.1 | JBJYMX000000000 | SAMN45706868 | EcoHS11212-879 |
| GCA_046246005.1 | JBJYMU000000000 | SAMN45706871 | EcoMHE1212-939 |
| GCA_046245985.1 | JBJYMV000000000 | SAMN45706870 | EcoHS11212-881 |
| GCA_046245925.1 | JBJYMT000000000 | SAMN45706872 | EcoMHE1212-940 |
| GCA_046245965.1 | JBJYMS000000000 | SAMN45706873 | EcoMHE1212-941 |
| GCA_046245935.1 | JBJYMR000000000 | SAMN45706874 | EcoMHE1212-944 |
| GCA_046245895.1 | JBJYMP000000000 | SAMN45706876 | EcoMHE1212-946 |
| GCA_046245885.1 | JBJYMQ000000000 | SAMN45706875 | EcoMHE1212-945 |
| GCA_046245845.1 | JBJYMN000000000 | SAMN45706878 | EcoMHE1212-948 |
| GCA_046245855.1 | JBJYMO000000000 | SAMN45706877 | EcoMHE1212-947 |
| GCA_046245795.1 | JBJYMK000000000 | SAMN45706881 | EcoMHE1801-951 |
| GCA_046245785.1 | JBJYML000000000 | SAMN45706880 | EcoMHE1801-950 |
| GCA_046245805.1 | JBJYMM000000000 | SAMN45706879 | EcoMHE1212-949 |
| GCA_046245765.1 | JBJYMJ000000000 | SAMN45706882 | EcoMHE1801-952 |
| GCA_046245735.1 | JBJYMH000000000 | SAMN45706884 | EcoMHE1801-955 |
| GCA_046245725.1 | JBJYMI000000000 | SAMN45706883 | EcoMHE1801-953 |
| GCA_046245695.1 | JBJYMG000000000 | SAMN45706885 | EcoMHE1801-956 |
| GCA_046245675.1 | JBJYMF000000000 | SAMN45706886 | EcoMHE1801-957 |
| GCA_046245665.1 | JBJYMD000000000 | SAMN45706888 | EcoSS2501-959 |
| GCA_046245645.1 | JBJYME000000000 | SAMN45706887 | EcoSS2501-958 |
| GCA_046245625.1 | JBJYMC000000000 | SAMN45706889 | EcoSS2501-960 |
| GCA_046245605.1 | JBJYMB000000000 | SAMN45706890 | EcoSS2501-961 |
| GCA_046245585.1 | JBJYLZ000000000 | SAMN45706892 | EcoSS2501-963 |
| GCA_046245565.1 | JBJYMA000000000 | SAMN45706891 | EcoSS2501-962 |
| GCA_046245545.1 | JBJYLX000000000 | SAMN45706894 | EcoSS2501-965 |
| GCA_046245515.1 | JBJYLY000000000 | SAMN45706893 | EcoSS2501-964 |
| GCA_046245465.1 | JBJYLT000000000 | SAMN45706898 | 687AMP0.32 |
| GCA_046245505.1 | JBJYLW000000000 | SAMN45706895 | EcoSS2501-966 |
| GCA_046245475.1 | JBJYLV000000000 | SAMN45706896 | EcoSS2501-967 |
| GCA_046245415.1 | JBJYLU000000000 | SAMN45706897 | 687-N |
| GCA_046245435.1 | JBJYLR000000000 | SAMN45706900 | 687CLOX64 |
| GCA_046245405.1 | JBJYLS000000000 | SAMN45706899 | 687AMP8 |
| GCA_046245335.1 | JBJYLM000000000 | SAMN45706905 | 956AMP8 |
| GCA_046245385.1 | JBJYLQ000000000 | SAMN45706901 | 687CLOX128 |
| GCA_046245325.1 | JBJYLN000000000 | SAMN45706904 | 956-N |
| GCA_046245315.1 | JBJYLP000000000 | SAMN45706902 | 876AMP8 |
| GCA_046245305.1 | JBJYLO000000000 | SAMN45706903 | 876CAZ0.25 |
| GCA_046245255.1 | JBJYLK000000000 | SAMN45706907 | 956CLOX25.6 |
| GCA_046245245.1 | JBJYLJ000000000 | SAMN45706908 | 956CLOX128 |
| GCA_046245215.1 | JBJYLL000000000 | SAMN45706906 | 956AMP16 |
| GCA_046245235.1 | JBJYLH000000000 | SAMN45706910 | 961CLOX64 |
| GCA_046245205.1 | JBJYLI000000000 | SAMN45706909 | 961CLOX25.6 |

| **Table S5: Antibiotics for which resistant results were obtained from the MIC assays of the 39 isolates in *bla*_CTX_ group that included AMP, CAZ, CTX, CPD, CFQ, ATM and TET.** | | | | | | | | | | | | | |
| --- | --- | --- | --- | --- | --- | --- | --- | --- | --- | --- | --- | --- | --- |
| **Antibiotics** | **AMP** | | **CAZ** | | **CTX** | | **CPD** | | **CFQ** | | **ATM** | | **TET** |
| **Breakpoints** | **Sensitive < / Resistant > (mg L^-1^)** | | | | | | | | | | | | |
| **EUCAST** | 8 | | 1/4 | | 1/2 | | 1 | | - | | 1/4 | | 4/16 |
| **Literature Stated** | - | | - | | - | | - | | 0.25 | | - | | - |
| **ECOFF** | 8 | | 0.5 | | 0.25 | | 1 | | 0.125 | | 0.25 | | - |
| **Isolates** | **MIC** | | | | | | | | | | | | |
| **687** | >512 | | 16 | | >512 | | 512 | | 128 | | 32 | | 64 |
| **726** | >512 | | 16 | | >512 | | 512 | | 128 | | 32 | | 64 |
| **774** | >512 | | 16 | | >512 | | 512 | | 128 | | 32 | | 64 |
| **873** | >512 | | 16 | | >512 | | 512 | | 128 | | 32 | | 64 |
| **874** | >512 | | 16 | | >512 | | 512 | | 128 | | 32 | | 128 |
| **875** | >512 | | 16 | | >512 | | 512 | | 128 | | 32 | | 128 |
| **876** | >512 | | 16 | | >512 | | 512 | | 128 | | 32 | | 128 |
| **877** | >512 | | 16 | | >512 | | 512 | | 128 | | 32 | | 64 |
| **878** | >512 | | 16 | | >512 | | 512 | | 128 | | 32 | | 64 |
| **879** | >512 | | 16 | | >512 | | 512 | | 128 | | 32 | | 64 |
| **880** | >512 | | 16 | | >512 | | 512 | | 128 | | 32 | | 128 |
| **881** | >512 | | 16 | | >512 | | 512 | | 128 | | 32 | | 128 |
| **939** | >512 | | 16 | | >512 | | 512 | | 128 | | 32 | | 64 |
| **940** | >512 | 16 | | >512 | | 512 | | 128 | | 32 | | 64 | |
| **941** | >512 | 16 | | >512 | | 512 | | 128 | | 32 | | 64 | |
| **942** | >512 | 16 | | >512 | | 512 | | 128 | | 32 | | 64 | |
| **944** | >512 | 16 | | >512 | | 512 | | 128 | | 32 | | 64 | |
| **945** | >512 | 16 | | >512 | | 512 | | 128 | | 32 | | 64 | |
| **946** | >512 | 16 | | >512 | | 512 | | 128 | | 32 | | 64 | |
| **947** | >512 | 16 | | >512 | | 512 | | 128 | | 32 | | 64 | |
| **948** | >512 | 16 | | >512 | | 512 | | 128 | | 32 | | 64 | |
| **949** | >512 | 16 | | >512 | | 512 | | 128 | | 32 | | 64 | |
| **950** | >512 | 16 | | >512 | | 512 | | 128 | | 16 | | 2 | |
| **951** | >512 | 16 | | >512 | | 512 | | 128 | | 16 | | 64 | |
| **952** | >512 | 16 | | >512 | | 512 | | 128 | | 16 | | 64 | |
| **953** | >512 | 16 | | >512 | | 512 | | 128 | | 32 | | <2 | |
| **955** | >512 | 16 | | >512 | | 512 | | 128 | | 16 | | <2 | |
| **956** | >512 | 16 | | >512 | | 512 | | 128 | | 32 | | <2 | |
| **957** | >512 | 16 | | >512 | | 512 | | 128 | | 16 | | 64 | |
| **958** | >512 | 16 | | >512 | | 512 | | 128 | | 32 | | 64 | |
| **959** | >512 | 16 | | >512 | | 512 | | 128 | | 32 | | 64 | |
| **960** | >512 | 16 | | >512 | | 512 | | 128 | | 32 | | 64 | |
| **961** | >512 | 16 | | >512 | | 512 | | 128 | | 32 | | 64 | |
| **962** | >512 | 16 | | >512 | | 512 | | 128 | | 32 | | 64 | |
| **963** | >512 | 16 | | >512 | | 512 | | 128 | | 32 | | 64 | |
| **964** | >512 | 16 | | >512 | | 512 | | 128 | | 32 | | 128 | |
| **965** | >512 | 16 | | >512 | | 512 | | 128 | | 32 | | 128 | |
| **966** | >512 | 16 | | >512 | | 512 | | 128 | | 32 | | 64 | |
| **967** | >512 | 16 | | >512 | | 512 | | 128 | | 32 | | 64 | |
| **ATCC25922** | <4 | <0.5 | | <0.25 | | 0.5 | | <0.25 | | <0.25 | | <2 | |
| ***Footnote for Table S5****: Any literature utilised breakpoints are written in red with the references to the literature used for obtaining these breakpoints*  *detailed as follows: CFG (Zhang et al. 2021). Any susceptible results are highlighted in green.* | | | | | | | | | | | | | |

| **Table S6: Antibiotics for which only susceptible results were obtained from the MIC assays of the 39 isolates in the *bla*_CTX_ group. As all 39 isolates returned identical MIC results, only the antibiotics, the EUCAST or literature-stated breakpoints (in red text) and the MIC result are listed.** | | |
| --- | --- | --- |
| **Antibiotic** | **EUCAST Breakpoints**  **Sensitive < / Resistant > (mg/L)** | **Result** |
| AMC | 8 | <4 |
| FOX | 8/32 | 2 |
| IMP | 2/4 | <0.5 |
| ERT | 0.5 | <0.032 |
| MER | 2/8 | <0.064 |
| STREP | 8/64 | 8 |
| GENT | 2 | <1 |
| NEO | 8/16 | <4 |
| APR | 8/64 | <4 |
| TOB | 2 | <1 |
| TIG | 0.5 | <0.25 |
| NAL | 32 | 32 |
| CIP | 0.25/0.5 | 0.25 |
| ENR | 2 | 1 |
| NIT | 64 | <32 |
| CHLOR | 8 | <4 |
| SXT | 2/4 | <0.5 |
| COL | 2 | <1 |
| AZM | 32 | <8 |
| ***Footnote for Table S6****: Any breakpoints in red text were literature-stated breakpoints with the references to the literature used for obtaining these breakpoints detailed as follows NAL (Ruiz et al. 2002), ENR (Temmerman et al. 2020) and AZM (Gomes et al. 2019).* | | |

**Table S7: Full meta data for all 105 ST2325 *E. coli* isolates downloaded from Enterobase and used in the phylogenetic tree analysis.**

***Footnote for Table S7****: US – United States, UK – United Kingdom, LUX – Luxembourg, NTL – Netherlands, GER – Germany, CAN – Canada, SGP – Singapore, AUS –*

*Australia, SA – South Africa, ND – Non-Defined, NA – Not Available*

| **Table S8: Assembly statistics for the 39 sequenced *bla*_CTX_ isolates (Bioproject:** [PRJNA736866](https://www.ncbi.nlm.nih.gov/portal/utils/pageresolver.fcgi?recordid=66b9ed79480a57696b4b8ea6))**.** | | | | | | | | |
| --- | --- | --- | --- | --- | --- | --- | --- | --- |
| **Isolate** | **Number of ORFs** | **Total Number of Contigs** | **Contig Number Containing a Plasmid (replicon and plasmid size (bp))** | **Complete Plasmid** | **Overall Genome Size (bp)** | **Overall %GC Content** | **N50 Number** | **Complete Chromosome** |
| 687 | 4,552 | 11 | 1. (IncFIC 61,878 bp)   3 (IncI2 59,595 bp) | Yes  Yes | 4,845,732 bp | 50.6% | 4,695,066 bp | Yes |
| 726 | 4,596 | 7 | 1. (IncI1 105,560 bp)   3 (IncF1C 61,868 bp) | Yes  Yes | 4,898,234 bp | 50.8% | 4,711,679 bp | Yes |
| 774 | 4,381 | 3 | None | n/a | 4,707,327 bp | 50.8% | 4,699,350 bp | Yes |
| 873 | 4,612 | 9 | 1. (IncI1 69,389 bp) 2. (IncFIC 61,867 bp)   4 (IncX4 32,452 bp) | Yes  Yes  Yes | 4,895,634 bp | 50.7% | 4,699,571 bp | Yes |
| 874 | 4,639 | 8 | 1. (IncI1 105,558 bp) 2. (IncFIC 62,755 bp)   4 (IncX4 32,450 bp) | Yes  Yes  Yes | 4,919,530 bp | 50.7% | 4,699,609 bp | Yes |
| 875 | 4,626 | 8 | 1. (IncI1 105,566 bp) 2. (IncFIC 61,867 bp)   4 (IncX4 32,451 bp) | Yes  Yes  Yes | 4,914,272 bp | 50.7% | 4,700,406 bp | Yes |
| 876 | 4,633 | 7 | 1. (IncI1 105,561 bp) 2. (IncFII 64,447 bp)   4 (IncX4 32,452 bp) | Yes  Yes  Yes | 4,914,853 bp | 50.7% | 4,700,179 bp | Yes |
| 877 | 4,623 | 9 | 1. (IncI1 87,563 bp) 2. (IncFIC 61,864 bp)   4 (IncX4 32,450 bp) | Yes  Yes  Yes | 4,910,607 bp | 50.7% | 4,709,599 bp | Yes |
| 878 | 4,619 | 17 | None | n/a | 4,806,936 bp | 50.8% | 3,900,948 bp | No |
| 879 | 4,338 | 8 | 1. (IncI1 84,267 bp) 2. (IncFIC 61,866 bp)   4 (IncX4 32,452 bp) | Yes  Yes  Yes | 4,855,001 bp | 50.7% | 4,658,078 bp | Yes |
| 880 | 4,601 | 24 | 5 (IncFIC/I1 50,117 bp)  6 (IncFIC/I1 47,504 bp)  7 (IncFIC/I1 42,967 bp)  8 (IncX4 32,451 bp) | No – IncFIC and IncI1 plasmids spread across contigs 5, 6 and 7  Yes | 4,890,733 bp | 50.7% | 1,545,096 bp | No |
| 881 | 4,622 | 8 | 1. (IncI1 102,050 bp) 2. (IncFIC 61,864 bp)   4 (IncX4 32,406 bp) | Yes  Yes  Yes | 4,910,094 bp | 50.7% | 4,910,094 bp | Yes |
| 939 | 4,595 | 5 | 1. (IncI1 105,564 bp)   3 (IncFIC 61,867 bp) | Yes  Yes | 4,896,868 bp | 50.7% | 4,721,460 bp | Yes |
| 940 | 4,584 | 5 | 1. (IncI1 105,565 bp)   3 (IncFIC 61,867 bp) | Yes  Yes | 4,886,824 bp | 50.7% | 4,711,415 bp | Yes |
| 941 | 4,597 | 5 | 1. (IncI1 105,564 bp)   3 (IncFIC 61,867 bp) | Yes  Yes | 4,897,401 bp | 50.7% | 4,721,993 bp | Yes |
| 942 | 4,514 | 4 | None | n/a | 4,751,314 bp | 50.7% | 4,656,624 bp | Yes |
| 944 | 4,625 | 7 | 1. (IncI1 105,565 bp) 2. (IncFIC 61,867 bp)   4 (IncX4 32,452 bp) | Yes  Yes  Yes | 4,912,049 bp | 50.7% | 4,699,951 bp | Yes |
| 945 | 4,612 | 8 | 1. (IncI1 105,565 bp)   3 (IncFIC 61,867 bp) | Yes  Yes | 4,911,418 bp | 50.7% | 4,721,662 bp | Yes |
| 946 | 4,612 | 8 | 1. (IncI1 105,563 bp)   3 (IncFIC 61,867 bp) | Yes  Yes | 4,911,410 bp | 50.7% | 4,721,657 bp | Yes |
| 947 | 4,625 | 7 | 1. (IncI1 105,564 bp) 2. (IncFIC 61,867 bp)   4 (IncX4 32,451 bp) | Yes  Yes  Yes | 4,910,848 bp | 50.7% | 4,699,875 bp | Yes |
| 948 | 4,478 | 4 | 2 (IncFIC 61,866 bp) | Yes | 4,791,830 bp | 50.8% | 4,721,987 bp | Yes |
| 949 | 4,613 | 7 | 1. (IncI1 105,565 bp)   3 (IncFIC 61,867 bp) | Yes  Yes | 4,915,453 bp | 50.7% | 4,732,465 bp | Yes |
| 950 | 4,593 | 5 | 1. (IncI1 105,565 bp)   3 (IncFIC 61,867 bp) | Yes  Yes | 4,891,432 bp | 50.7% | 4,716,023 bp | Yes |
| 951 | 4,606 | 5 | 1. (IncI1 105,566 bp)   3 (IncFIC 61,867 bp) | Yes  Yes | 4,907,351 bp | 50.7% | 4,731,941 bp | Yes |
| 952 | 4,602 | 6 | 1. (IncI1 105,563 bp)   3 (IncFIC 61,866 bp) | Yes  Yes | 4,902,828 bp | 50.7% | 4,722,079 bp | Yes |
| 953 | 4,587 | 6 | 1. (IncI1 105,557 bp)   3 (IncFIC 61,850 bp) | Yes  Yes | 4,881,647 bp | 50.7% | 4,695,262 bp | Yes |
| 955 | 4,594 | 5 | 1. (IncI1 105,559 bp)   3 (IncFIC 61,867 bp) | Yes  Yes | 4,892,035 bp | 50.7% | 4,716,632 bp | Yes |
| 956 | 4,589 | 6 | 1. (IncI1 105,562 bp)   3 (IncFIC 61,867 bp) | Yes  Yes | 4,893,273 bp | 50.7% | 4,716,660 bp | Yes |
| 957 | 4,599 | 6 | 1. (IncI1 105,562 bp)   3 (IncFIC 61,866 bp) | Yes  Yes | 4,899,970 bp | 50.7% | 4,722,983 bp | Yes |
| 958 | 4,412 | 5 | 2 (IncFIC 61,866 bp) | Yes | 4,735,971 bp | 50.8% | 4,702,390 bp | Yes |
| 959 | 4,647 | 6 | 1. (IncI1 105,558 bp) 2. (IncFIC 61,863 bp)   4 (IncX4 39,090 bp) | Yes  Yes  Yes | 4,928,079 bp | 50.7% | 4,713,591 bp | Yes |
| 960 | 4,386 | 8 | 1. (IncI1 105,562 bp) 2. (IncFIC 61,865 bp)   4 (IncX4 39,095 bp) | Yes  Yes  Yes | 4,929,909 bp | 50.7% | 4,712,431 bp | Yes |
| 961 | 4,629 | 5 | 1. (158,269 bp) 2. (IncX4 39,095 bp) | Yes but IncI1 and IncFIC both in contig 2  Yes | 4,919,354 bp | 50.7% | 4,714,013 bp | Yes |
| 962 | 4,628 | 9 | 1. (75,590 bp) 2. (51,134 bp)   5 (24,952 bp)  4 (IncX4 39,064 bp) | No IncI1 and IncFIC spread across contigs 2, 3 and 5  Yes | 4,915,154 bp | 50.7% | 4,708,700 bp | Yes |
| 963 | 4,518 | 7 | 1. (IncFIC 61,865 bp)   3 (IncI1 40,261 bp) | Yes  Yes | 4,825,533 bp | 50.7% | 4,711,319 bp | Yes |
| 964 | 4,590 | 7 | 1. (IncFIC 61,864 bp)   3 (IncX4 39,095 bp) | Yes  Yes | 4,869,976 bp | 50.7% | 4,723,307 bp | Yes |
| 965 | 4,458 | 6 | 2 (IncFIC 45,492 bp) | Yes | 4,769,443 bp | 50.7% | 4,711,498 bp | Yes |
| 966 | 4,558 | 7 | 2 (IncI1 105,560 bp) | Yes | 4,860,469 bp | 50.7% | 4,720,358 bp | Yes |
| 967 | 4,592 | 9 | 1. (IncFIC 61,865 bp) 2. (IncI1 51,220 bp)   4 (IncX4 39,094 bp) | Yes  Yes  Yes | 4,874,260 bp | 50.7% | 4,704,796 bp | Yes |
| ***Footnote for Table S8****: The assembly statistics included the number of ORFS, total number of contigs, contig number containing a plasmid with plasmid replicon and size (bp), whether the plasmid was complete, overall genome size (bp), %GC content, N50 number and whether chromosome was complete.* | | | | | | | | |

| **Table S9: Group 1 TTs with IS*Ecp1* elements >15 kb in length.** | | | | | | | | | |
| --- | --- | --- | --- | --- | --- | --- | --- | --- | --- |
| **TT Name** | | **Plasmid Replicon Type Used in Conjugation** | | **IS*Ecp1* Size** | **Insertion Point in Plasmid** | | | **IR_R(new2)_ utilised by IS*Ecp1* with bases complementary to the IR_L_ shown in red and number of bases complementary to the IRL detailed underneath** | |
| 687CLOX128 | | IncFIC | | 27,093 bp | Interrupted *traC* and recognition of a new imperfect IR_R_ took additional genes from T3SS during transfer | | | 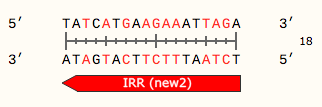  **10 bases complementary to the IR_L_** | |
| 961CLOX64 | | IncI1 | | 27,093 bp | Truncated a Hypothetical Protein (HP) and inserted between T1 TA System *hok*/*gef* Toxin and a gene encoding a HP. Recognition of a new imperfect IR_R_ took additional genes from T3SS during transfer | | | 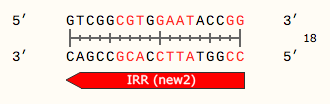  **9 bases complementary to the IR_L_** | |
| 687AMP0.32 | | IncFIC | | 27,094 bp | Interrupted a gene encoding a Hypothetical Protein (HP) and recognition of a new imperfect IR_R_ took additional genes from T3SS during transfer | | | 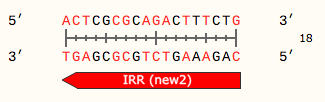  **11 bases complementary to the IR_L_** | |
| 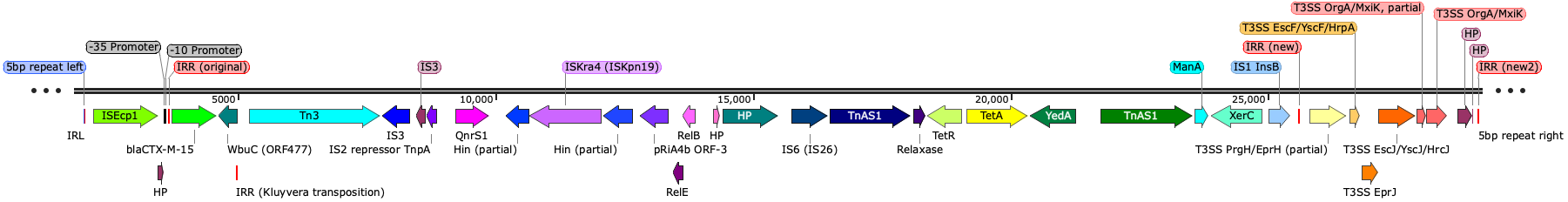 | | | | | | | | | |
| ***Footnote for Table S9****: Detailed is the plasmid replicon type use in the conjugative transfer of ISEcp1, the insertion point of ISEcp1 in the plasmid, the IRR sequence used with complementary base to IR_L_ shown below and a small graphic of the ISEcp1 element in each TT* | | | | | | | | | |
| **Table S10: Group 2 TTs where IS*Ecp1* elements were all between 10-15 kb in length.** | | | | | | | | | |
| **TT Name** | | **Plasmid Replicon Type Used in Conjugation** | | | **IS*Ecp1* Size** | **Insertion Point in Plasmid** | | **IR_R(new2)_ utilised by IS*Ecp1* with bases complementary to the IR_L_ shown in red and number of bases complementary to the IR_L_ detailed underneath** | |
| 687-N | | IncFIC | | | 11,394 bp | Interrupted an Alpha/Beta Hydrolase gene and inserted near *traX* and *finO.* Recognition of a new imperfect IR_R_ resulted in the loss of Tn*AS1* region encoding *tetAR*. | | 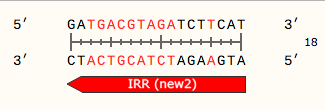  **10 bases complementary to the IR_L_** | |
| 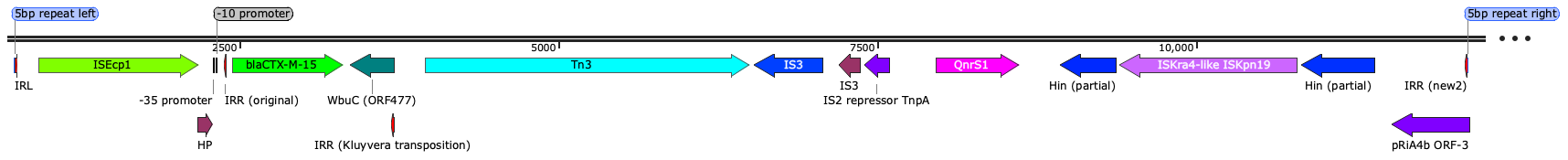 | | | | | | | | | |
| 956AMP8 | | IncFIC | | | 11,394 bp | No interrupted genes but inserted between a gene encoding a DUF2726 and *finO* | | 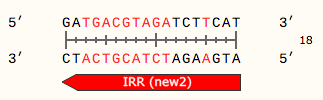  **10 bases complementary to the IR_L_** | |
| 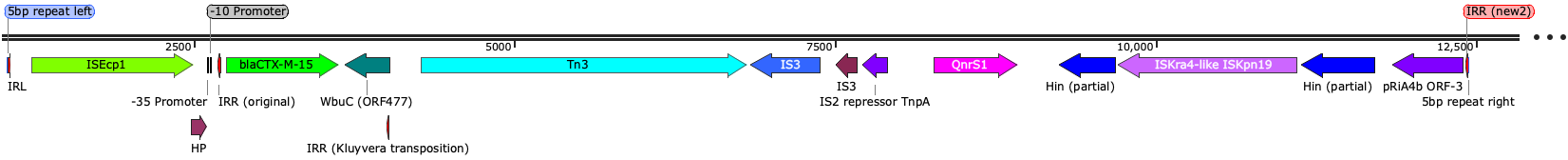 | | | | | | | | | |
| 956AMP16 | | IncFIC | | | 12,196 bp | No interrupted genes but inserted between genes encoding a TIR Domain and a Plasmid Stabilization Protein | | 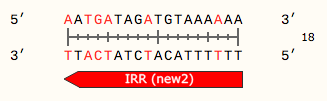  **6 bases complementary to the IR_L_** | |
| 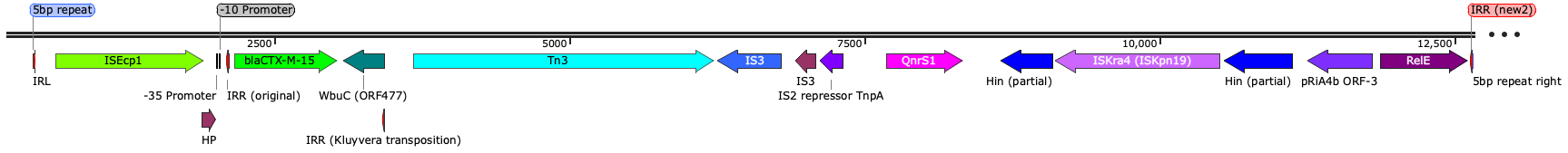 | | | | | | | | | |
| 961CLOX25.6 | | IncX4 | | | 14,722 bp | Truncated Tn*AS1* and Interrupted *virB8.* Recognition of a new imperfect IR_R_ resulted in the loss of *tetAR*. | | 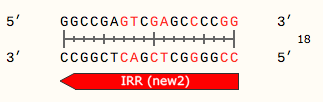  **7 bases complementary to the IR_L_** | |
| 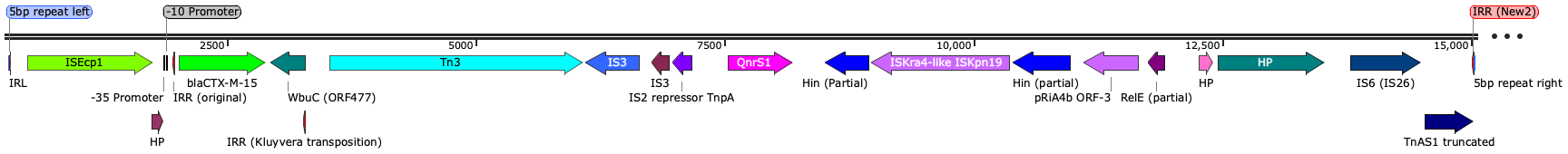 | | | | | | | | | |
| ***Footnote for Table S10****: Detailed is the plasmid replicon type use in the conjugative transfer of ISEcp1, the insertion point of ISEcp1 in the plasmid, the IRR sequence used with complementary base to IRL shown below and a small graphic of the ISEcp1 element in each TT* | | | | | | | | | |

| **Table S11: Group 3 TTs where IS*Ecp1* elements were all between <10 kb in length.** | | | | |
| --- | --- | --- | --- | --- |
| **TT Name** | **Plasmid Used by IS*Ecp1*** | **IS*Ecp1* Size** | **Truncated genes and insertion point in plasmid** | **IR_R(new2)_ utilised by IS*Ecp1* with bases complementary to the IR_L_ shown in red and detailed underneath** |
| 687AMP8 | IncFIC | 2,706 bp | Truncated *wbuC* and inserted between IncFII *repA* and a gene encoding a DUF2726. Recognition of a new imperfect IR_R_ resulted in the loss of all genes beyond *wbuC*, that included the Tn*3*, IS*3*, *qnrS1*, the two partial halves of *hin*, IS*Kra4*, *relEB*, IS*6* and the Tn*AS1* region encoding *tetAR*, *manA* and *xerC* | 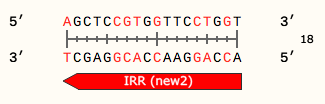  **8 bases complementary to the IR_L_** |
| 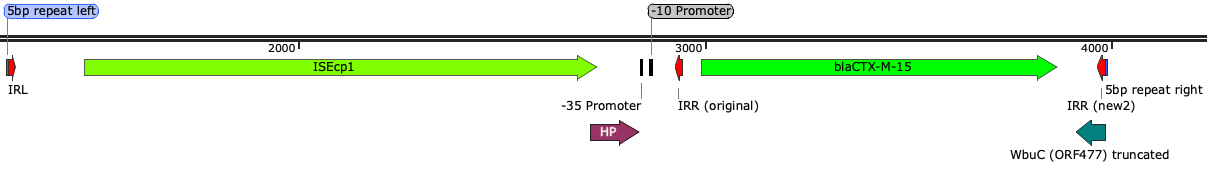 | | | | |
| 687CLOX64 | IncFIC | 8,284 bp | No interrupted genes but insertion was between *traI* and *traX*. Recognition of a new imperfect IR_R_ resulted in the loss of all genes beyond *qnrS1* that included the two partial halves of h*in*, IS*Kra4*, *relEB*, IS*6* and the Tn*AS1* region encoding *tetAR*, *manA* and *xerC* | 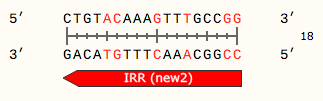  **6 bases complementary to the IR_L_** |
| 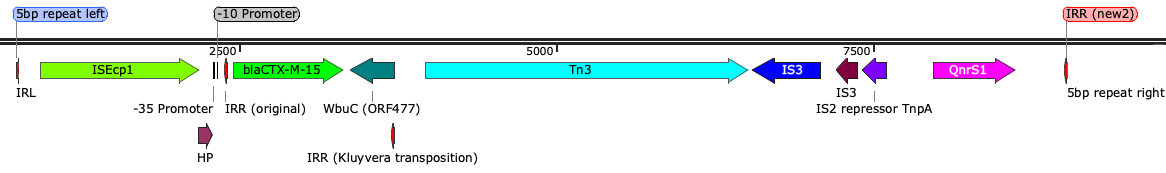 | | | | |
| 876AMP8 | IncI1 | 2,706 bp | Truncated *wbuC* and interrupted *traX.* Recognition of a new imperfect IR_R_ resulted in the loss of all genes beyond *wbuC*, that included the Tn*3*, IS*3*, *qnrS1*, the two partial halves of *hin*, IS*Kra4*, *relEB*, IS*6* and the Tn*AS1* region encoding *tetAR*, *manA* and *xerC* | 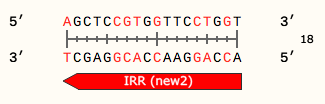  **8 bases complementary to the IR_L_** |
| 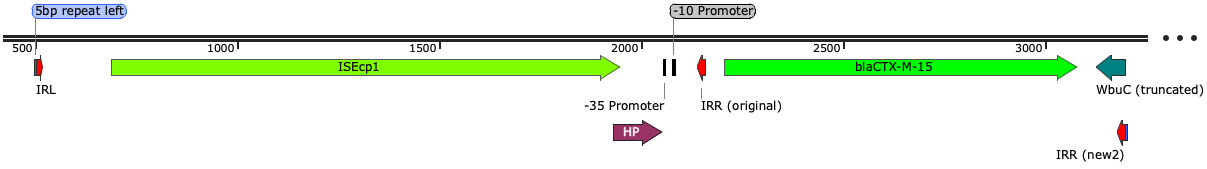 | | | | |
| 876CAZ0.25 | IncFII | 3,172 bp | Truncated *traD* by inserting adjacent to the stop codon TGA, resulting in readthrough of *traD.* Recognition of a new imperfect IR_R_ resulted in the loss of all genes beyond *wbuC*, that included the Tn*3*, IS*3*, *qnrS1*, the two partial halves of *hin*, IS*Kra4*, *relEB*, IS*6* and the Tn*AS1* region encoding *tetAR*, *manA* and *xerC* | 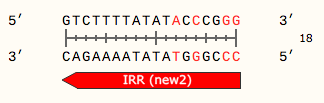  **4 bases complementary to the IR_L_** |
| 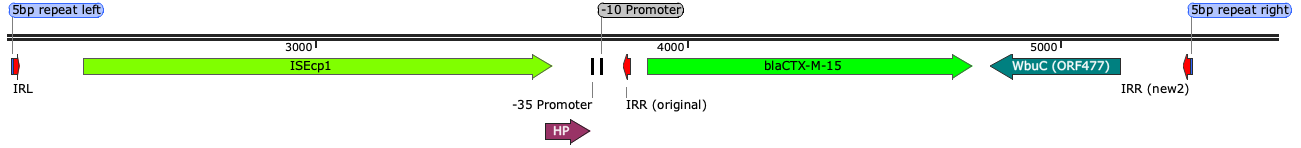 | | | | |
| 956-N | IncFIC | 2,981 bp | Truncated *wbuC* and used IR_R(_*_Kluyvera_* _transposition)_ during transfer. Recognition of a new imperfect IR_R_ resulted in the loss of all genes beyond *wbuC*, that included the Tn*3*, IS*3*, *qnrS1*, the two partial halves of *hin*, IS*Kra4*, *relEB*, IS*6* and the Tn*AS1* region encoding *tetAR* | 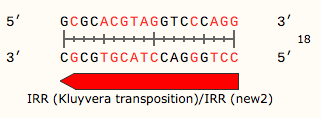  **11 bases complementary to the IR_L_** |
| 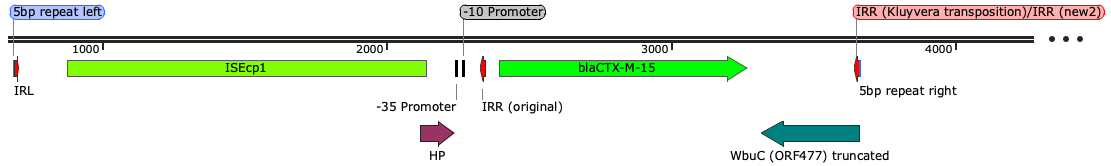 | | | | |
| 956CLOX25.6 | IncFIC | 3,060 bp | Interrupted an Alpha/Beta Hydrolase and inserted near *traX* and *finO.* Recognition of a new imperfect IR_R_ resulted in the loss of all genes beyond *wbuC*, that included the Tn*3*, IS*3*, *qnrS1*, the two partial halves of *hin*, IS*Kra4*, *relEB*, IS*6* and the Tn*AS1* region encoding *tetAR* | 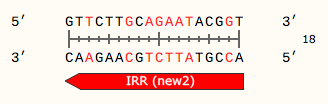  **8 bases complementary to the IR_L_** |
| 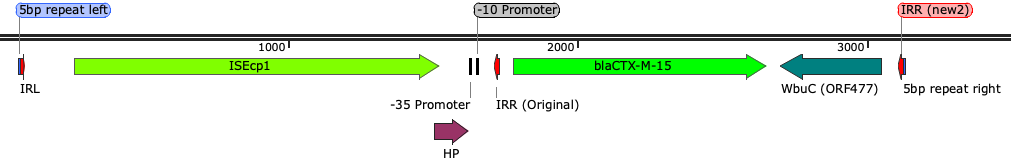 | | | | |
| 956CLOX128 | IncFIC | 2,981 bp | No interrupted genes but inserted between an Alpha/Beta hydrolase gene and *finO*. Used IR_R(_*_Kluyvera_* _transposition)_ during transfer. Recognition of a new imperfect IR_R_ resulted in the loss of all genes beyond *wbuC*, that included the Tn*3*, IS*3*, *qnrS1*, the two partial halves of *hin*, IS*Kra4*, *relEB*, IS*6* and the Tn*AS1* region encoding *tetAR* | 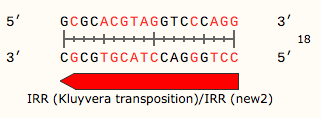  **11 bases complementary to the IR_L_** |
| 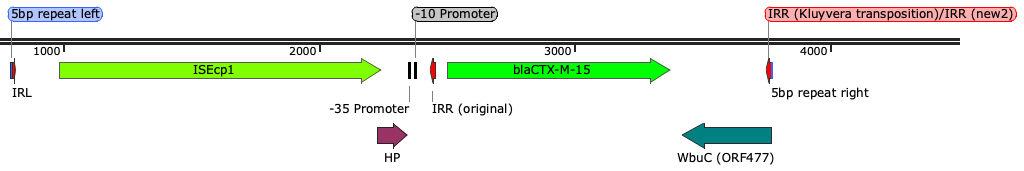 | | | | |
| ***Footnote for Table S11****: Detailed is the plasmid replicon type use in the conjugative transfer of ISEcp1, the insertion point of ISEcp1 in the plasmid, the IRR sequence used with complementary base to IRL shown below and a small graphic of the ISEcp1 element in each TT* | | | | |

**Supplementary References**

1. Baker, M., Williams, A.D., Hooton, S.P.T., Helliwell, R., King, E., Dodsworth, T., María Baena-Nogueras, R., Warry, A., Ortori, C.A., Todman, H., Gray-Hammerton, C.J., Pritchard, A.C.W., Iles, E., Cook, R., Emes, R.D., Jones, M.A., Kypraios, T., West, H., Barrett, D.A., Ramsden, S.J., Gomes, R.L., Hudson, C., Millard, A.D., Raman, S., Morris, C., Dodd, C.E.R., Kreft, J.U., Hobman, J.L. and Stekel, D.J. 2022. Antimicrobial resistance in dairy slurry tanks: A critical point for measurement and control. *Environment International* 169, p. 107516.
2. Gomes, C., Ruiz-Roldán, L., Mateu, J., Ochoa, T.J. and Ruiz, J. 2019. Azithromycin resistance levels and mechanisms in *Escherichia coli*. *Scientific Reports* 9(1).
3. Ruiz, J. et al. 2002. High prevalence of nalidixic acid resistant, ciprofloxacin susceptible phenotype among clinical isolates of *Escherichia coli* and other *Enterobacteriaceae*. *Diagnostic Microbiology and Infectious Disease* 42(4), pp. 257–261. doi: 10.1016/S0732-8893(01)00357-1.
4. Sgro, G.G., Oka, G.U., Souza, D.P., Cenens, W., Bayer-Santos, E., Matsuyama, B.Y., Bueno, N.F., Dos Santos, T.R., Alvarez-Martinez, C.E., Salinas, R.K. and Farah, C.S. 2019. Bacteria-killing type IV secretion systems. *Frontiers in Microbiology* 10(MAY), p. 1078. doi: 10.3389/fmicb.2019.01078.
5. Temmerman, R., Garmyn, A., Antonissen, G., Vanantwerpen, G., Vanrobaeys, M., Haesebrouck, F. and Devreese, M. 2020. Evaluation of Fluoroquinolone Resistance in Clinical Avian Pathogenic *Escherichia coli* Isolates from Flanders (Belgium). *Antibiotics* 9(11), pp. 1–14.
6. Zhang, H. lin, Zhao, Y. yang, Zhou, Z. chong and Ding, H. zhong. 2021. Susceptibility breakpoint for cefquinome against *Escherichia coli* and *Staphylococcus aureus* from pigs. *Journal of Integrative Agriculture* 20(7), pp. 1921–1932. doi: 10.1016/S2095-3119(20)63572-9.
